# Apoptosis-inducing anti-HER2 agents operate through oligomerization-induced receptor immobilization

**DOI:** 10.1038/s42003-021-02253-4

**Published:** 2021-06-21

**Authors:** Jakob C. Stüber, Christian P. Richter, Junel Sotolongo Bellón, Martin Schwill, Iwo König, Benjamin Schuler, Jacob Piehler, Andreas Plückthun

**Affiliations:** 1 Department of Biochemistry, University of Zurich, Zurich, Switzerland; 2 Department of Biology/Chemistry and Center for Cellular Nanoanalytics, Osnabrück University, Osnabrück, Germany; 3 Present Address: Roche Pharma Research & Early Development, Large Molecule Research, Roche Innovation Center Munich, Penzberg, Germany; 4 Present Address: Roche Diagnostics Int. AG, Rotkreuz, Switzerland

**Keywords:** Tumour biomarkers, Biophysical chemistry, Biological fluorescence, Supramolecular assembly, Recombinant protein therapy

## Abstract

Overexpression of the receptor tyrosine kinase HER2 plays a critical role in the development of various tumors. Biparatopic designed ankyrin repeat proteins (bipDARPins) potently induce apoptosis in HER2-addicted breast cancer cell lines. Here, we have investigated how the spatiotemporal receptor organization at the cell surface is modulated by these agents and is distinguished from other molecules, which do not elicit apoptosis. Binding of conventional antibodies is accompanied by moderate reduction of receptor mobility, in agreement with HER2 being dimerized by the bivalent IgG. In contrast, the most potent apoptosis-inducing bipDARPins lead to a dramatic arrest of HER2. Dual-color single-molecule tracking revealed that the HER2 “lockdown” by these bipDARPins is caused by the formation of HER2-DARPin oligomer chains, which are trapped in nanoscopic membrane domains. Our findings establish that efficient neutralization of receptor tyrosine kinase signaling can be achieved through intermolecular bipDARPin crosslinking alone, resulting in inactivated, locked-down bipDARPin-HER2 complexes.

## Abstract (web summary)

Stüber et. al. show that receptor tyrosine kinase signaling can be neutralized through intermolecular, biparatopic bipDARPin crosslinking that locks down the bipDARPin-HER2 complexes. This affects the spatiotemporal organization and dynamics of HER2 in the plasma membrane.

## Introduction

Human epidermal growth factor receptor 2 (HER2/ErbB2/neu) has gained tremendous importance as a biomarker in diagnostics and a target in the therapy of breast cancers, but also in gastric and gastroesophageal cancers, over the past decade^1^. This receptor tyrosine kinase has a distinct role within the ErbB family of receptors, in that it has no ligand. Deduced from the rigidity seen in all crystal structures, in molecular dynamics, and binding experiments, HER2 has therefore been assumed to always be in a dimerization-competent state^2^. Under physiological conditions, HER2 likely does not homodimerize, and thus its role is restricted to that of a signal-amplifying co-receptor for other, ligand-activated members of its family^3^. Upon malignant overexpression, however, spontaneous formation of homodimers and heterodimers, even in the absence of ligands of the other ErbB family member, enables sustained proliferation and survival signaling^4^. This stimulus, in turn, frequently transforms the cancer cell signaling architecture towards singular dependence on HER2 signaling^5^. Such HER2-addicted cancers are more aggressive but, at the same time, also susceptible to targeted therapies. Therefore, HER2 overexpression, previously merely a correlate of bad prognosis, nowadays generally also implies the availability of several targeted treatment options.

Among these, monoclonal antibodies (mAbs) have been successfully developed that bind to the HER2 extracellular domain and thus block activity. Prominent examples that have been approved for therapy are the humanized mouse mAbs trastuzumab (TZB) and pertuzumab (PZB)^1^. Interestingly, the mechanisms of HER2 inhibition by TZB and PZB are complementary (targeting complexes with the non-liganded and liganded states of HER3, respectively), and therefore most efficacious therapies employ the combination of both mAbs^6,7^. Of note, subtle changes in the relative orientation of the Fab domains can transform F(ab’)_2_-like molecules derived from TZB into active pro-proliferative agents, underlining the importance of binding geometry for the modulation of HER2 activity by affinity reagents^8^.

**Fig. 1:**
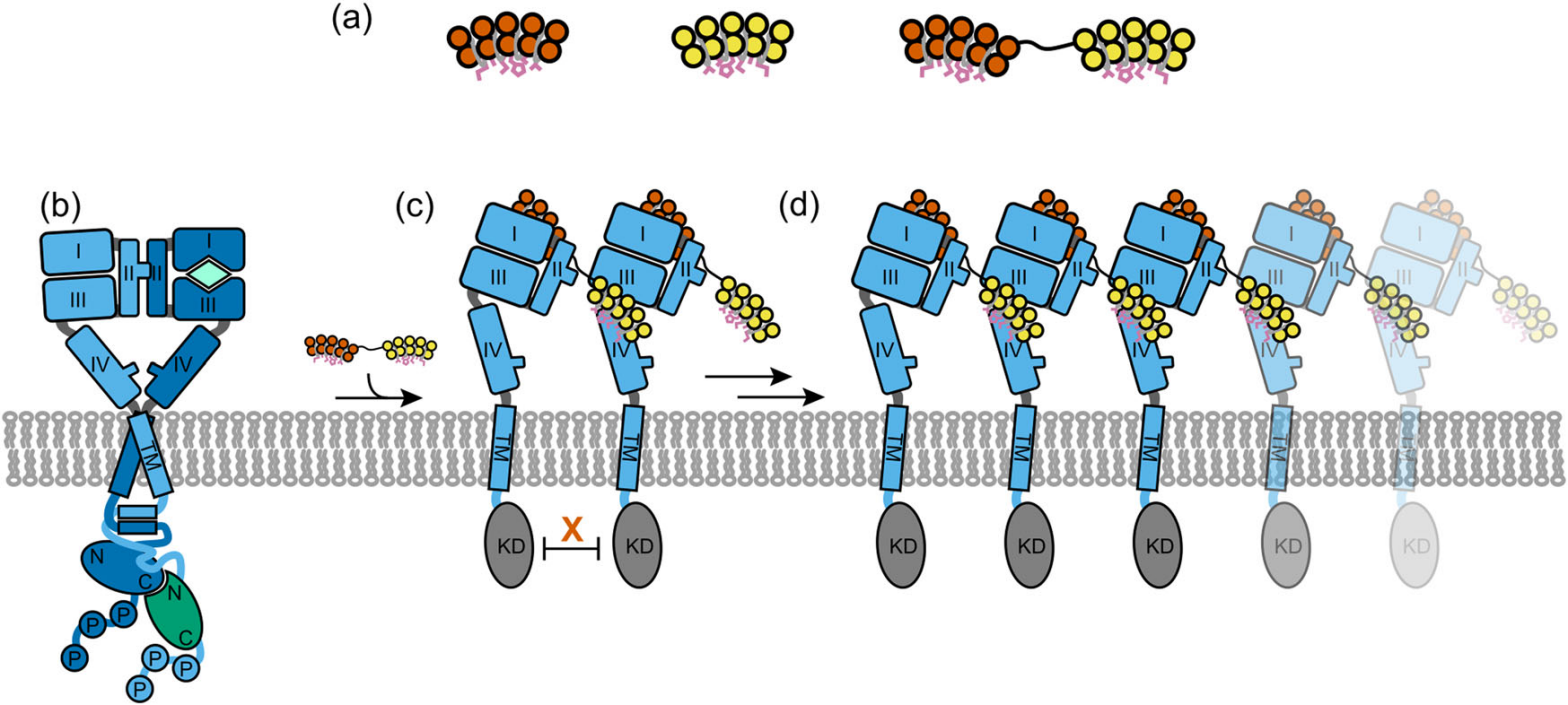
Concept of bipDARPins and possible modes of binding to HER2. **a** Monovalent, monoparatopic DARPins (9.26 binding to extracellular subdomain I, vermillion; G3 binding to extracellular subdomain IV, yellow) and design of bipDARPin 6L1G (right), connecting 9.26 and G3 through a short linker. **b** Ligand-activated (e.g., by heregulin, mint) HER2:HER3 (sky blue, blue) heterodimers are able to stimulate proliferation and survival. **c**, **d** Bivalent intermolecular binding of bipDARPins may induce the formation of (**c**) inactive dimers or (**d**) oligomerization into daisy chains. In both cases, the kinase domains are separated and thus inactive. HRG heregulin, TM transmembrane helix, KD kinase domain.

Recently, we have reported a novel strategy based on designed ankyrin repeat proteins (DARPins)^9^ to inhibit HER2 activity in breast cancer cells^10,11^. To this end, we engineered biparatopic antitumor DARPins (bipDARPins) consisting of two binding moieties, which recognize the extracellular subdomains I and IV of HER2, respectively (Fig. 1a). These binding moieties are connected by a short linker (see Supplementary Fig. 1 for a detailed overview), with the aim to trap and stabilize an inactive conformation^11^. Compared to TZB and PZB, bipDARPins much more efficaciously promote apoptosis in HER2-dependent tumor cell lines^10^ by dephosphorylating HER2 (and not only HER3) and subsequently preventing re-activation of phosphoinositide-3 kinase through RAS^10^. While the signaling pathways underlying this effect have been characterized in detail^10^, the exact mechanism responsible for the potent inhibitory function of bipDARPins, in contrast to less active molecules, has so far remained largely unclear. Less active molecules not only include the antibodies TZB and PZB but also the same DARPin units with longer linker in between or in different orientation^10,11^.

X-ray structures of single DARPin moieties in complex with the isolated, soluble extracellular subdomains of HER2^11^ led to the conclusion that the most active bipDARPins cannot bind both epitopes in an intramolecular fashion, implying that rather two neighboring HER2 molecules are engaged (Fig. 1c, d). In contrast, intramolecular trapping would be possible for the less active molecules with longer linkers. Possible mechanisms of action considered had initially included locking an inactive HER2 conformation similar to the tethered conformation seen in the other ErbB receptors, but we have shown subsequently that the extracellular domain is very rigid, making such a conformation highly unlikely^2^. This suggests that inhibition is instead achieved via restrained dimerization (Fig.1c, d), in which interactions between the cytosolic tyrosine kinase domains required for allosteric activation are prevented^12^. However, this binding mode also may allow elongation into daisy chain-like arrangements (Fig.1d), which we have previously proposed to cause efficient inhibition of HER2 signaling^11^.

To delineate the mechanism responsible for HER2 inhibition by bipDARPins, we explored here their effects on the spatiotemporal organization and dynamics of HER2 in the plasma membrane. Chemical crosslinking in combination with immunoprecipitation revealed oligomerization induced by active bipDARPins, but not by TZB and PZB. Making use of cell surface-specific posttranslational labeling techniques, we quantified the diffusion properties of HER2 in the plasma membrane of living cells by fluorescence recovery after photobleaching (FRAP) and by single-molecule localization microscopy (SMLM). Efficient arrest of HER2 diffusion was observed upon treatment with bipDARPins, while control experiments with TZB and PZB yielded only a slight reduction in mobility. Dual-color single-molecule tracking (SMT) and co-tracking demonstrate that bipDARPins induce clustering and immobilization of HER2 at the cell surface. Detailed analyses of local diffusion properties uncovered transient nanoscale immobilization, potentially caused by entrapment in plasma membrane subcompartments. These findings presented here strongly suggest that the signaling-incompetent states of HER2, induced by bipDARPin binding, are signaling-inactive oligomers formed directly through crosslinking. In addicted cell lines, this state leads to pan-HER inhibition^10^.

## Results

### Chemical crosslinking suggests that bipDARPins induce HER2 oligomer formation

**Fig. 2:**
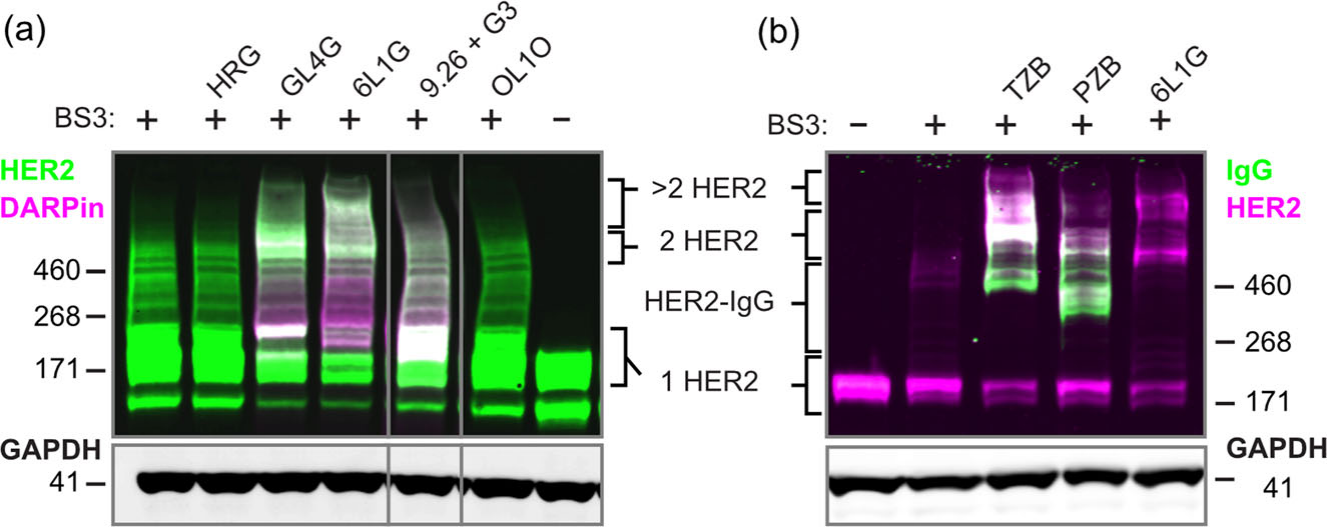
Oligomerization of HER2 (~180 kDa) by bipDARPins and antibodies on BT474 cells analyzed by chemical crosslinking and two-color western blot. **a** Comparison of the size of HER2 complexes after treatment with the bipDARPin 6L1G (32 kDa) with those obtained with growth factor heregulin (HRG, 27 kDa), monovalent DARPins (9.26 and G3, ~12 kDa each) or a bivalent, monoparatopic DARPin (GL4G, ~30 kDa). Primary antibodies against HER2 and DARPins were used. **b** Crosslinked HER2 complexes upon treatment with antibodies trastuzumab (TZB, ~145 kDa) and pertuzumab (PZB, ~145 kDa), detected by primary antibodies against HER2 and human IgG.

To characterize the stoichiometries of HER2 complexes formed with bipDARPins, we performed chemical crosslinking analyses with bis[sulfosuccinimidyl]suberate (BS3) using BT474 cells, a model for HER2-addicted breast cancer^10^ with a highly elevated expression level of approximately 9 × 10^5^ copies of HER2 per cell^13^. In the absence of treatment, as well as after addition of the HER3-binding ligand HRG, we primarily detected non-crosslinked (monomeric) HER2, and only minor nonspecific crosslinking adducts (Fig. 2a). As expected, the homo-bivalent monoparatopic control DARPin GL4G (Supplementary Fig. 1) led to a band at the expected size of two crosslinked HER2 molecules. Remarkably, the high-potency bipDARPin 6L1G not only yielded a similar band but also an additional band staining for both HER2 and DARPin at a size corresponding to complexes of 3–4 HER2 molecules. Control treatments, either with the monovalent moieties contained within 6L1G, DARPins 9.26 and G3, the combination of these monovalent DARPins, or with the non-HER2-binding control DARPin OL1O (that binds bacterial maltose binding protein) did not alter the band pattern observed for HER2 alone.

We also investigated HER2 crosslinking with BS3 after incubation with TZB or PZB (Fig. 2b). As previously described^14^, TZB seemed to be preferably crosslinked, forming a ternary complex with two HER2 molecules, running at a higher apparent size than the presumed HER2 dimer without TZB. In contrast, PZB seemed not to lead to an increase in crosslinked species that would contain two or more HER2 molecules. Instead, HER2 and PZB appeared to be preferably interconnected in a 1:1 ratio. Together, these results suggested that bipDARPins not only dimerized HER2 in an inactive state but also induced daisy chain formation as depicted in Fig. 1f.

### FRAP measurements reveal immobilization of HER2 by bipDARPins

To further explore bipDARPin-induced oligomerization of HER2 suggested by these crosslinking experiments, we probed receptor diffusion dynamics at the surface of live cells by single-molecule and ensemble fluorescence imaging techniques. To ensure selective labeling of proteins at the cell surface with photostable fluorophores, we fused HER2 to an N-terminal HaloTag^15^ (HT-HER2), which was reacted with membrane-impermeable, negatively charged dyes conjugated to the HaloTag ligand (HTL). Thus, the variation of labeling density and optimal dual-color labeling was readily achieved by adjusting the labeling conditions, which allowed us to apply and compare different imaging techniques using the same HER2 expression construct and total cell-surface expression levels. As we included a long, flexible linker between the receptor’s N-terminus and the HaloTag, interference with any receptor function is minimized^16^.

**Fig. 3:**
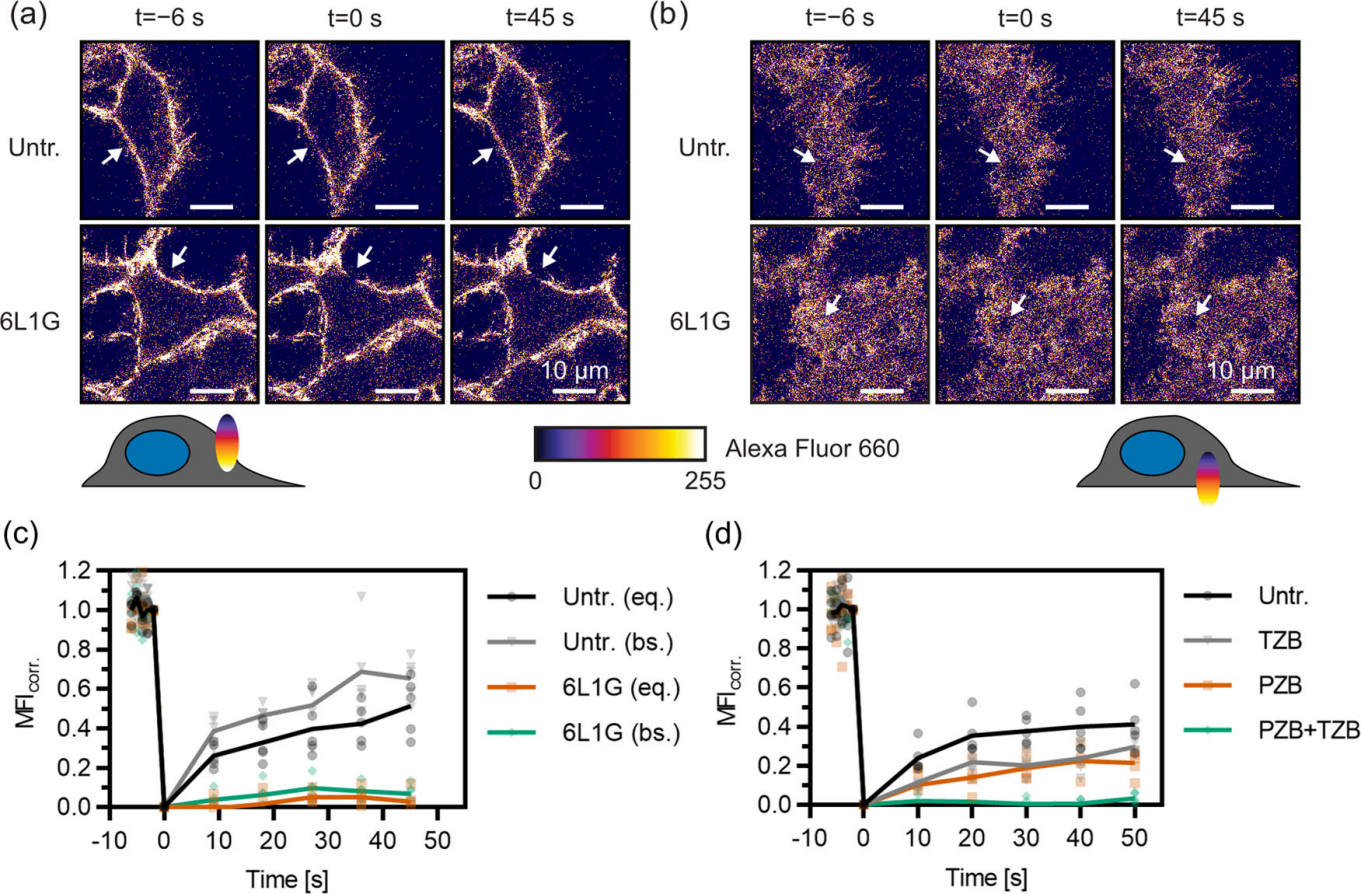
Changes in the mobility of Halo-tagged HER2 on the surface of HEK293 cells upon treatment with DARPins and mAbs, quantified by FRAP. **a**, **b** Confocal images from a representative FRAP experiment of HT-HER2 labeled with Alexa Fluor 660, by photobleaching either **a** in the equatorial (eq.) or **b** basal (bs.) membrane plane. The arrow indicates the location of the bleach spot (2 µm diameter). **c**, **d** FRAP curves (bleach-corrected, normalized mean fluorescence intensities in the bleached ROI after subtraction of residual intensity at *t* = 0) for various DARPin and antibody treatments and controls. **c** Massive reduction of mobility upon treatment with DARPin 6L1G. Data from the basal membrane are shown in addition to those obtained by regular measurements in the equatorial plane. **d** Slight reduction in mobility is seen upon treatment with the therapeutic mAbs PZB and TZB individually but complete immobilization by their combination (PZB + TZB). Untr., untreated.

To obtain an overall picture of receptor mobility in the plasma membrane, we used the classic FRAP technique^17^ with densely labeled HT-HER2. For this purpose, we stably transfected HEK293-derived T-Rex cells, which are based on the Flp-In system for inducible overexpression, with HT-HER2. To exclude a bias from receptor endocytosis and recycling, FRAP experiments were typically performed under conditions of ATP depletion^18^. Photobleaching was applied to a circular spot of 2 µm in diameter. We measured in the equatorial plane rather than at the basal membrane^19^, because a better signal-to-noise ratio could be achieved due to spatial integration over more molecules in the *z*-dimension (Fig. 3a, b). Photobleaching was performed in a single scan, yielding a decrease by 40–60% compared to the pre-bleach fluorescence intensity, while photobleaching during subsequent acquisition of fluorescence recovery was negligible. As the resulting distorted geometry of the photobleached volume within the membrane precludes application of established analytical models for fitting the recovery curves^20,21^, we directly compared the FRAP curves obtained under different conditions, as suggested elsewhere^19^.

**Fig. 4:**
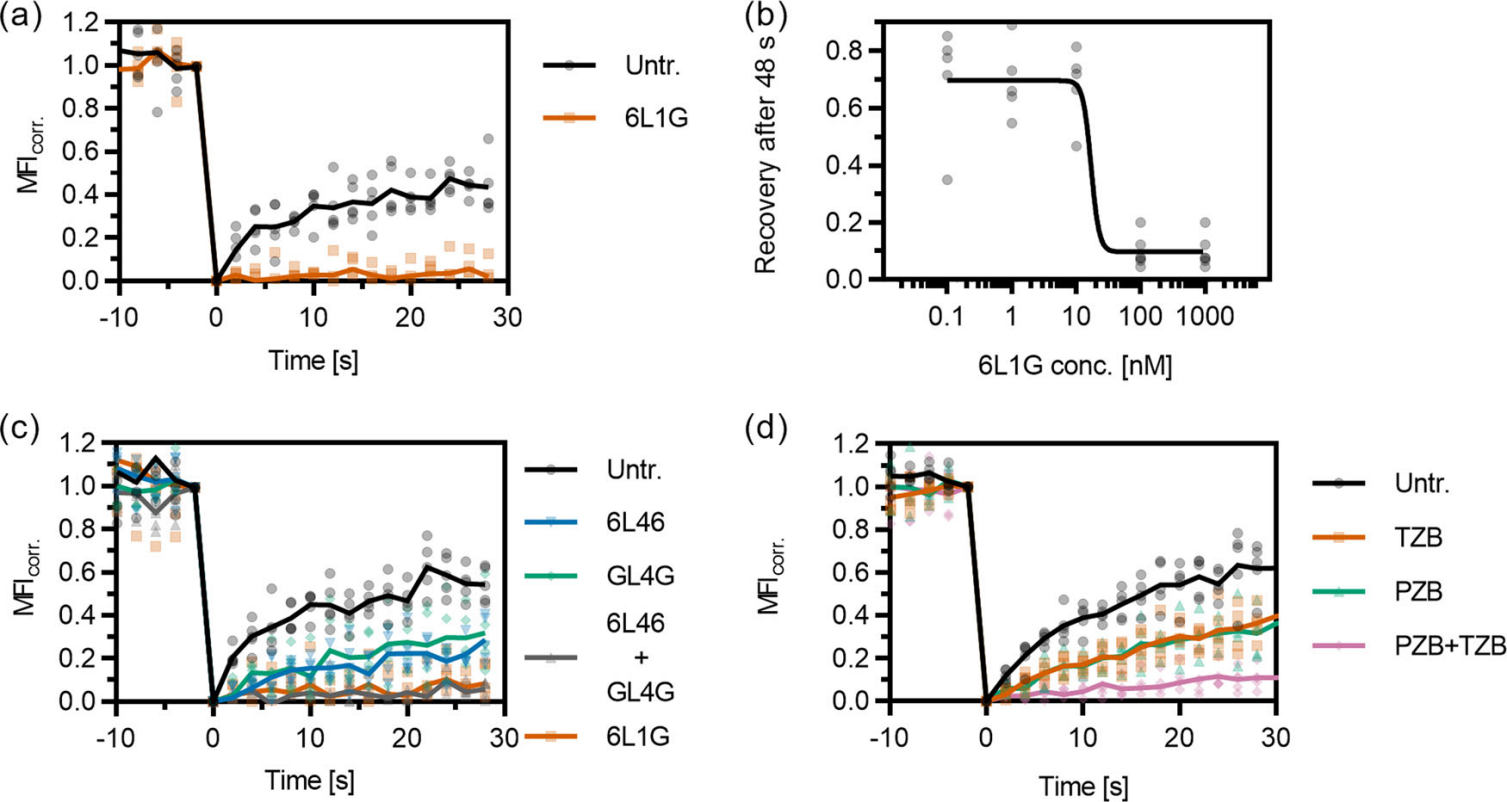
Mobility changes of endogenous HER2 on the surface of BT-474 cells (labeled via the affibody ZHER2) upon treatment with DARPins and mAbs as quantified by FRAP. **a** Dramatic loss of mobility upon treatment with bipDARPin 6L1G compared to untreated cells (Untr.). **b** Concentration dependence of the mobility reduction as illustrated by the relative fluorescence recovery after 48 s. **c** Homo-bivalent, monoparatopic DARPins 6L46 and GL4G reduce HER2 mobility only slightly, while a mixture of both (GL4G + 6L46) fully immobilizes HER2 through crosslinking. **d** Similarly, moderate mobility changes are observed upon treatment with therapeutic mAbs PZB and TZB but almost complete immobilization by their combination (PZB + TZB).

**Fig. 5:**
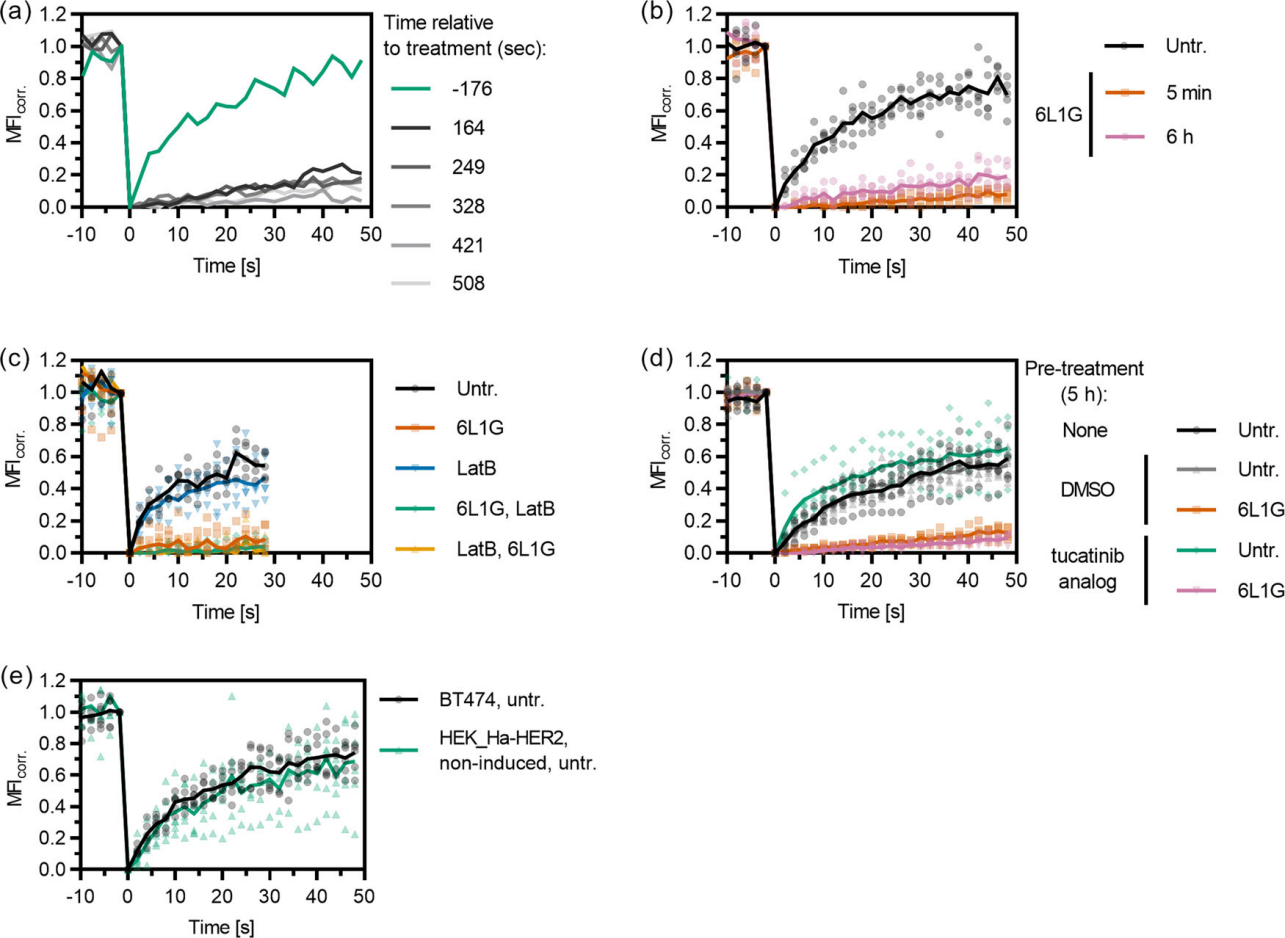
Immediate and sustained immobilization of HER2 (labeled by the affibody ZHER2) on the surface of BT-474 cells is mediated through DARPin–receptor interactions. **a** Individual FRAP curves (no averaging) before and after addition of DARPin 6L1G, colored according to the starting time relative to DARPin addition, show immediate immobilization. **b** HER2 immobilization is similar after 5 min and 6 h of treatment. **c** FRAP experiments in cells treated with Latrunculin B in the absence of DARPins (LatB), and cells treated with LatB before (LatB, 6L1G) or after (6L1G, LatB) bipDARPin addition indicate that cytoskeleton interactions are not directly involved in HER2 immobilization upon DARPin treatment. **d** Pre-treatment with a HER2-specific HER2 kinase inhibitor (Selleckchem S2752, an analog of ARRY 380) does not affect immobilization, suggesting that immobilization is independent of HER2 kinase activation. **e** HER2 immobilization occurs at a wide range of expression levels, as indicated by FRAP measurements on HER2-overexpressing BT-474 cells and non-induced HEK cells stably transfected with HaloTagged HER2. Note that the sensitivity of the FRAP method limits the cell lines that can be investigated to those with at least intermediate expression levels (Supplementary Table 1 and Supplementary Fig. 2). Data for untreated cells (Untr.) and 6L1G in **c** are identical to those shown in Fig. 4c.

In the absence of treatment, we consistently observed only 40–50% maximum recovery of photobleached HER2 within the observation time of 30–50 s that was sufficient to reach a plateau of the FRAP curve (Figs. 3–5). Strikingly, dramatic reduction of HER2 mobility was observed upon treatment with active bipDARPins, with maximum recovery levels of <10% reached within the observation time (Fig. 3a, c). This “lockdown” of HER2 mobility by bipDARPins was also observed for FRAP experiments at the basal cell membrane (Fig. 3b, c). The homo-bivalent antibodies TZB and PZB, which presumably dimerize HER2, each reduced the rate and maximum level of HER2 recovery by ~50%. Remarkably, the combination of TZB and PZB also induced complete HER2 immobilization (Fig. 3d), in line with oligomerization that can be caused by alternating crosslinking with two mAbs of non-overlapping epitopes^19^.

### Efficient lockdown of endogenous HER2 by bipDARPins in breast cancer cells

To confirm the biological relevance of bipDARPin-induced lockdown of HER2, we employed the small (6 kDa) affibody Zher2 (ref. ^22^) for labeling endogenous, untagged HER2 in breast cancer cells. Zher2 binds epitopes that are non-overlapping with any of the used DARPins or antibodies^10,11,23^, thus ensuring minimum interference. We applied Zher2 for probing the receptor dynamics at the surface of BT-474 cells. FRAP experiments confirmed similar diffusion dynamics of Zher2-labeled HER2 in the plasma membrane of BT-474 cells as observed for HT-HER2 in TREx-HEK293 cells (Fig. 4a). Strikingly, strong lockdown of HER2 upon treatment with the biologically most active DARPin 6L1G was confirmed for the endogenous receptor (Fig. 4a), in a clearly dose-dependent manner (Fig. 4b).

In comparison, the monoparatopic, homo-bivalent controls 6L46 and GL4G both led to a similar, moderate reduction in mobility when applied individually. By contrast, a mixture of these two binders abolished HER2 mobility completely, in agreement with receptor oligomerization by crosslinking (Fig. 4b). Similarly, as seen for HT-HER2 on TREx-HEK cells, TZB or PZB slightly reduced the mobility of HER2, while their combination lead to immobilization. Thus, the HER2 complexes formed upon binding of the single bipDARPin 6L1G resemble those resulting from mixtures of two homo-bivalent, monoparatopic agents (DARPins or antibodies) with non-overlapping epitopes, rather than the single agents.

### BipDARPin-HER2 complex formation suffices for immobilization

HER2 immobilization occurred rapidly upon bipDARPin binding, as illustrated by several consecutive FRAP measurements before and after 6L1G addition (Fig. 5a). No significant increase in HER2 mobility was observed even 6 h after treatment (Fig. 5b), demonstrating that immobilization is not a transient effect based on an immediate cellular response to DARPin binding, but that it persists as long as 6L1G is present. This suggests that bipDARPins and HER2 rapidly (within the dead time of the experiment, ~160 s) form complexes, which remain at the cell surface for extended periods, in line with the long internalization half-life of HER2 (ref. ^24^) and stable surface and internal HER2 levels upon 6L1G treatment^25^.

To test whether the cortical membrane cytoskeleton contributed to HER2 lockdown, we performed FRAP experiments in the presence of Latrunculin B (LatB), which depolymerizes actin. However, LatB treatment neither affected HER2 mobility in untreated cells nor when added before or after treatment with 6L1G (Fig. 5c). Furthermore, treatment with a HER2-specific kinase inhibitor neither impacted the mobility of HER2 alone nor the ability of 6L1G to cause HER2 lockdown (Fig. 5d). This confirms that phosphorylation is not involved in HER2 immobilization. To test whether the high baseline fraction of intrinsically immobile HER2 was restricted to cell lines with extremely strong HER2 expression, we compared the results from BT-474 breast cancer cells (which strongly overexpress HER2) to those obtained from our TREx-HEK293 cell line without induction of HER2 overexpression, which results in intermediate expression levels (Supplementary Table 1 and Supplementary Fig. 2). The recovery curves appear virtually identical, again showing ~40% of HER2 being immobile on a ~50 s timescale (Fig. 5e).

### BipDARPins induce immobilization of HER2 at the single-molecule level

The strong loss of HER2 mobility identified by the FRAP experiments clearly supported our model that bipDARPins induce HER2 clustering into daisy chains. To further investigate the local diffusion properties of HER2, we used single-molecule techniques. First, we probed fluorescence fluctuations within a diffraction-limited confocal volume positioned on the apical plasma membrane, using low-density dual-color labeling of HT-HER2 with Cy3 and AlexaFluor 647 (AF647), respectively. In the absence of bipDARPins, distinct fluorescence bursts originating from fluorescently labeled HER2 molecules diffusing through the focus were observed after an initial bleaching phase to reach a steady state (Supplementary Fig. 3a, b). Strikingly, the number of observed bursts dramatically decreased after adding the biologically active bipDARPin 9L1G but not after adding the monovalent control DARPin OL1G (Supplementary Fig. 3b). Quantitative burst detection^26^ confirmed the loss of HER2 mobility upon treatment with bipDARPins (Supplementary Fig. 3c).

The strongly reduced number of bursts suggested that HER2 lockdown by bipDARPins reduces mobility at length scales on the order of the size of the confocal volume. To explore the spatiotemporal re-organization of HER2 by bipDARPins with sub-diffraction resolution and to directly visualize immobile HER2-DARPin complexes, we turned to SMLM. For this purpose, we transiently expressed HER2 fused to an N-terminal SNAP_f_ tag^27^ in HeLa cells. After simultaneous labeling with DY-549P1 and DY-649P1, we performed time-lapse dual-color total internal reflection fluorescence microscopy (TIRFM) for tracking and co-tracking individual HER2 in the basal plasma membrane of living cells. To ensure reliable single-molecule localization and tracking, cells with an observable particle density <1 µm^−2^ in each channel were selected (see below). Given an estimated labeling degree of 40% for the SNAP_f_ tag^28^ and the expected level of endogenous HER2 in HeLa cells (Supplementary Table 1 and Supplementary Fig. 2), the fraction of labeled HER2 was <10% in these experiments.

**Fig. 6:**
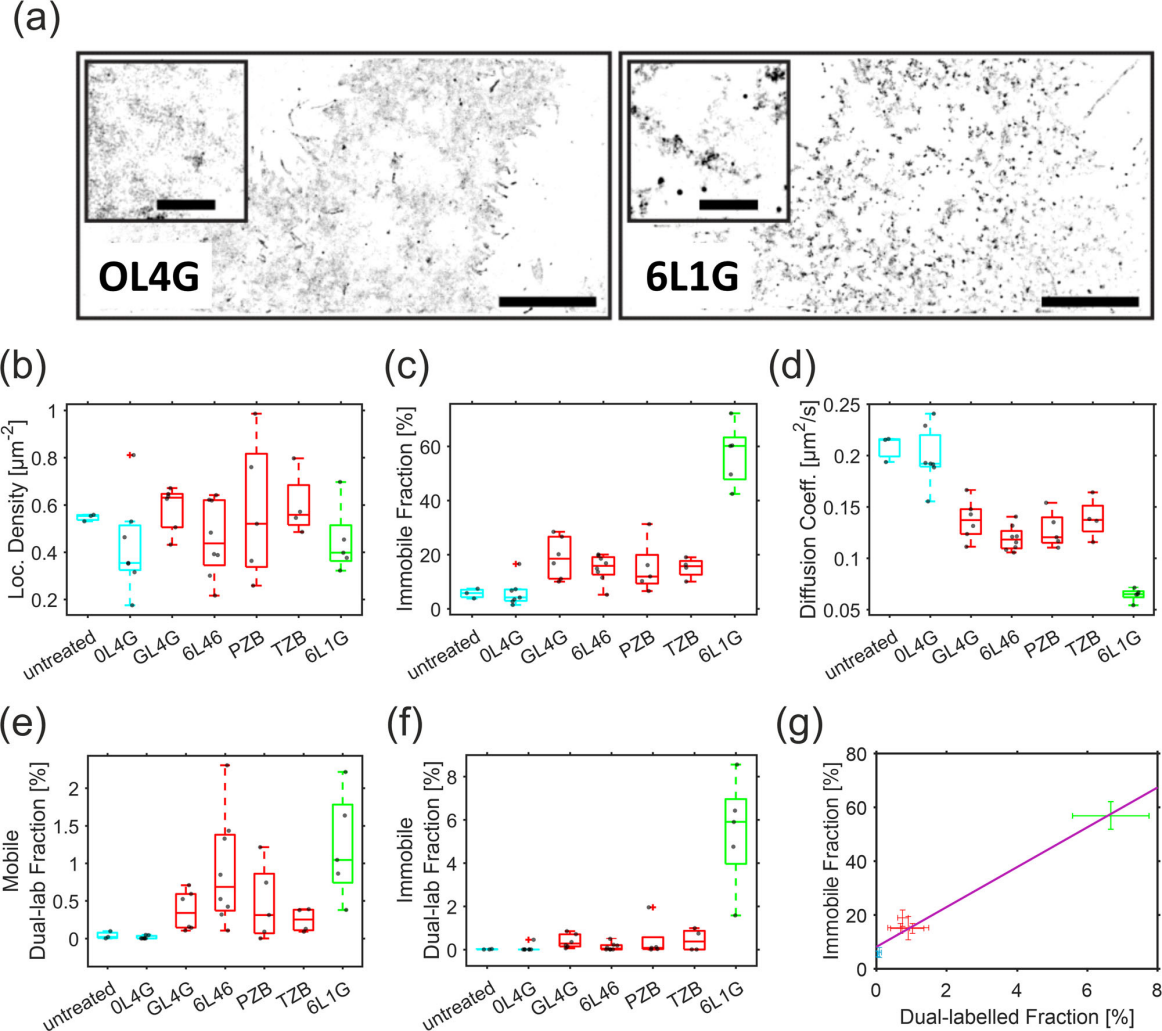
Lockdown and oligomerization of HER2 diffusion as resolved by single-molecule localization microscopy. **a** Representative SMLM super-resolution images rendered from 500 consecutive frames (localization precision ~20 nm) with localization densities encoded as gray values. Scale bar: 10 µm. Magnified center regions are shown as insets (scale bar: 2 µm). **b** Particle densities in the DY-649 channel observed in different single-molecule experiments. **c**, **d** Comparison of the immobile fraction identified by spatiotemporal clustering analysis (**c**) and the diffusion coefficient within the mobile fraction (**d**) for HER2 for different treatments. **e**, **f** Colocalized HER2 molecules in the mobile (**e**) and the immobile fraction (**f**) quantified by dual-color co-tracking. **g** Correlation of the total fraction of co-localized molecules with the immobile fraction and linear regression (R^2^ = 0.97, Pearson’s *r* = 0.99, *p* = 3.5 × 10^−5^). Different colors (cyan, control or monovalent; red, homo-bivalent; green, biparatopic) in **b**–**g** indicate the three distinct groups of agents as referenced in the main text. The DARPins are shown schematically in Supplementary Fig. 1. Significance values for **b**–**f** are provided in Supplementary Fig. 4.

To assess the spatiotemporal organization and dynamics of HER2, super-resolution images were rendered from single-molecule localizations obtained within 500 consecutive frames. These images revealed a strikingly speckled appearance for cells treated with the active bipDARPin 6L1G, compared to a monovalent control (Supplementary Movie 1, Supplementary Movie 2, and Fig. 6a). Since negligible photobleaching or photoblinking can be assumed during the observation time, the observed characteristics for HER2 in the presence of the active bipDARPin can be attributed to a dramatic loss in mobility, leading to repeated detection of the same molecule at the same location over time. By contrast, a much more spatially homogeneous distribution is observed in the negative control experiment in cells treated with the monovalent DARPin OL4G. Only few speckles were observed under these conditions, indicating largely unhindered diffusion.

We therefore analyzed in more detail the diffusion properties of HER2 molecules after treatment with different DARPins and mAbs, by SMT performed at comparable particle density to minimize bias (Fig. 6b, Supplementary Fig. 6b, and Supplementary Table 2). Immobile molecules were identified by a spatiotemporal clustering algorithm to quantify the immobile fraction (Fig. 6c and Supplementary Table 2). For the mobile fraction, an average diffusion constant was determined by mean squared displacement (MSD) analysis (Fig. 6d, Supplementary Fig. 5, and Supplementary Table 2). Comparison of these two parameters identified three distinct groups among the agents (Fig. 6c, d): (i) only a small immobile fraction (<10%) and high diffusion constant of the mobile fraction was found in the absence of treatment as well as for the monovalent control DARPin OL4G (cf. Supplementary Fig. 1); (ii) a slightly increased immobile fraction (15–20%) and a somewhat reduced mobility was found for homo-bivalent (monoparatopic) controls GL4G and 6L46 (Supplementary Fig. 1) and (likewise homo-bivalent, monoparatopic) antibodies TZB and PZB, all of which are expected to dimerize HER2; (iii) a markedly higher immobile fraction (>50%) was found for the biparatopic DARPin 6L1G, accompanied by a further decrease in the average diffusion constant of the mobile fraction.

### HER2 immobilization correlates with oligomerization

These observations suggested that diffusion behavior directly reflects the differential oligomerization properties. To verify this correlation, we exploited simultaneous dual-color detection for analyzing dimerization and oligomerization of HER2 by DARPins and mAbs with sub-diffraction resolution. To this end, co-tracking of molecules localized in both spectral channels was performed to unambiguously identify HER2 complexes comprising two or more molecules. The fraction of such co-trajectories observed after treatment with different binders are compared in Fig. 6e. Only negligible dimerization levels (<0.05%) were detected in control experiments without any agent and in cells treated with the monovalent DARPin OL4G, thus confirming a limited intrinsic tendency of HER2 to homodimerize^29^. By contrast, all other DARPins and mAbs significantly increased the fraction of co-locomoting HER2 molecules (Fig. 6e and Supplementary Table 2). Owing to the relatively low degree of labeling used in these experiments (<10%), however, these numbers cannot be directly converted into fractions of dimers and oligomers. Based on the *relative* numbers observed for different HER2 binders, however, their propensity to dimerize and oligomerize HER2 can be compared. Similar co-locomotion levels around 0.5% were observed for the homo-bivalent, monoparatopic DARPins 6L46 and GL4G as well as for TZB and PZB, all of which presumably dimerize HER2. A significantly higher value of 1.04% was observed for the bipDARPin 6L1G, indicating higher oligomerization levels, with an average of ~4 molecules/oligomer estimated from the ~2-fold increased dual-labeled fraction. Substantially stronger differences were observed for the immobile fraction. Here, the dual-labeled fractions for dimerizing agents were similar as for the mobile fractions, while it increased by a factor of ~6 for 6L1G (Fig. 6f and Supplementary Table 2). In line with this observation, we found a strong correlation of immobile and dual-labeled fraction size (Fig. 6g). Taken together, these results suggest that the bipDARPin 6L1G crosslinks HER2 into oligomers of different sizes (~4–10 copies), which is accompanied by a severe loss in mobility.

To confirm this hypothesis, we performed single-molecule localization, tracking, and co-tracking experiments with HER2 in the presence of the crosslinking mixture of 6L46 and GL4G. Under these conditions, largely identical spatiotemporal dynamics and diffusion of HER2 as compared to treatment with 6L1G were observed (Supplementary Figs. 6a, b and 7a). As expected, the crosslinking mixture of 6L46 and GL4G also induced a comparable increase of the immobile fraction, as well as a decrease of the average diffusion coefficient of the remaining mobile particles (Supplementary Figs. 6c, d and 7b, c). Likewise, very similar levels of dual-labeled mobile and immobile fractions were found in the presence of the 6L46–GL4G combination and 6L1G (Supplementary Figs. 6e, f and 7d, e), corroborating that similar levels of oligomerization are induced by these treatments.

### HER2 lockdown is caused by increased trapping in membrane nanodomains

**Fig. 7:**
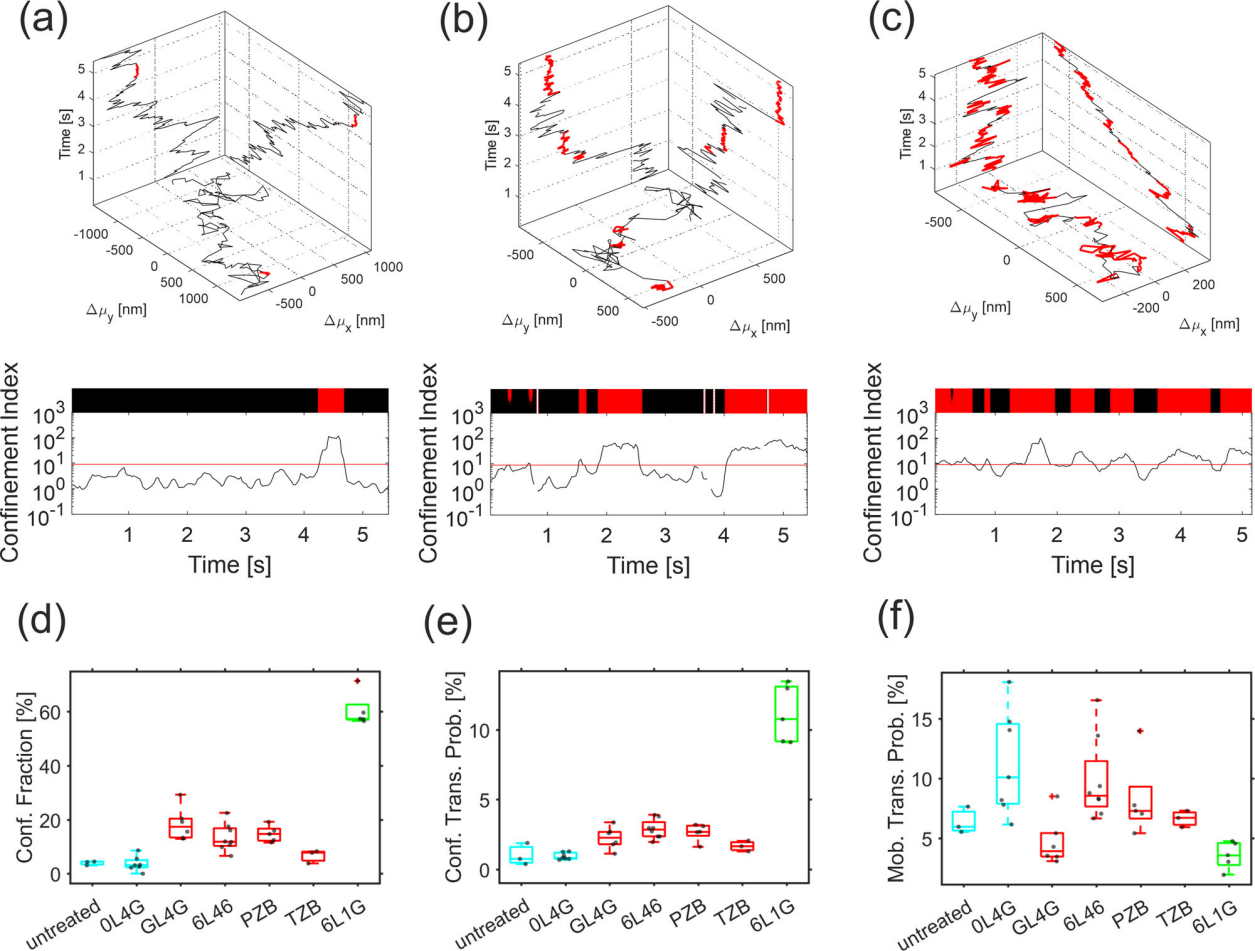
Lockdown is related to transient arrest of HER2. **a**, **b**, **c** Transient arrest events (highlighted in red) within individual HER2 trajectories (top) determined from the confinement index calculated along a representative trajectory (bottom) upon treatment with OL4G (**a**), GL4G (**b**), and 6L1G (**c**). The threshold defining transient arrest events is indicated by the red line. **d** Identified fraction of confined particles. **e** Probability of transitioning into state of arrested diffusion. **f** Probability of mobilization out of arrested diffusion. Different colors in **d**–**f** indicate the three distinct groups of agents as described in Fig. 6 and referenced in the main text. The DARPins are shown schematically in Supplementary Fig. 1. Significance values for **d**–**f** are provided in Supplementary Fig. 8.

Given the high mobility of HER2 in the absence of DARPins, the dramatic effect of dimerization and oligomerization on the diffusion remains surprising. We therefore more carefully analyzed the diffusion properties of HER2 by unbiased SMT, which was applied to cells with very low cell surface expression levels to ensure high tracking fidelity. Confinement analysis revealed characteristic short-term arrests of mobile HER2, which was occasionally observed even for untreated cells or after incubation with the monovalent OL4G (Fig. 7a–c, Supplementary Movies 3 and 4, and Supplementary Table 2). Upon treatment with mAbs and homo-bivalent DARPins that presumably dimerize HER2, an enhanced occurrence of such transient arrest events was observed, while the dwell times of the arrested state remained rather constant (Fig. 7d–f, Supplementary Fig. 9a, b, and Supplementary Table 2). Treatment with the apoptosis-inducing bipDARPin 6L1G strongly further enhanced the occurrence of HER2 transient arrest events but only moderately increased the dwell time of the arrested state. Thus, mobility within the 32-s time frame of these experiments was still observed for most HER2 molecules even after treatment with 6L1G, with HER2 essentially “dropping” from one confinement zone into the next (Supplementary Movie 4 and Supplementary Fig. 9e). Single-molecule trajectories of transiently immobilized particles revealed residual, highly confined diffusion (Supplementary Movies 5–7 and Supplementary Fig. 9e), suggesting transient trapping in nanoscopic membrane domains with a diameter of ~35 nm. Upon treatment with 6L1G, characteristic successive transient immobilization of HER2 was observed (Fig. 7c, Supplementary Movie 4, and Supplementary Fig. 9e). Taken together, these results suggest that the efficient “lockdown” of HER2 by bipDARPins does not involve irreversible immobilization but rather can be ascribed to a strongly increased propensity for being trapped within nanoscopic confinement zones.

## Discussion

Blocking the activity of cell surface receptor tyrosine kinases by mAbs and other binders has emerged as a highly successful strategy in cancer therapy, and frequently these agents block ligand-binding-mediated activation. Molecular mechanisms effective for inhibiting constitutive activation, however, have largely remained unclear. We have previously introduced bipDARPins that bind HER2 via two distinct epitopes, which yielded a surprisingly effective inhibition of downstream signaling pathways, leading to apoptosis in addicted cell lines and in vivo^10^. To pinpoint the mechanism responsible for HER2 inhibition, we have investigated here the properties of the HER2–bipDARPin complexes in the plasma membrane. We were particularly intrigued by the possibility that bipDARPins may inhibit HER2 at the plasma membrane by the formation of daisy chains (cf. Fig. 1d).

While chemical crosslinking confirmed that active bipDARPins crosslink HER2 into oligomers, the 3–4 copies of HER2 we found in these oligomers was surprisingly low. To resolve this conundrum, we focused on the spatiotemporal organization and dynamics of HER2 in the plasma membrane of live cells and its changes upon treatment with different binders. Remarkably, even in untreated cells, a substantial fraction of HER2 (40–60%) was immobile within the time and length scale of our FRAP experiments. Similar levels of immobile fractions have been previously observed in FRAP experiments with HER2, epidermal growth factor receptor (EGFR), and ErbB3 in a variety of cell lines^29–33^, suggesting that this may be a fundamental feature of ErbB family receptors. In line with previous reports^29,32^, depolymerizing the cortical actin cytoskeleton did not lead to any significant changes in mobility (Fig. 5c), suggesting that the HER2 immobilization is not caused by the membrane skeleton. SMT, however, revealed high local mobility of HER2 and residual diffusion even in the presence of bipDARPins. Rather, we identified transient immobilization even in the absence of any binder as a characteristic feature of HER2 diffusion in the plasma membrane. Such short-term immobilization has been related to receptor trapping in nanoscopic plasma membrane sub-compartments^34–36^, which have been reported for several cell surface receptors including the ErbB family members EGFR^32,37^ and HER2^32^. Our results suggest that such transient immobilization of HER2 may result into apparent local confinement when observed on the much larger length scale probed by FRAP.

Treatment with DARPins and mAbs that dimerize or oligomerize HER2 substantially enhanced the frequency of transient immobilization events, leading to a dramatic loss in overall HER2 mobility as observed by FRAP. Dimerizing DARPins and mAbs already increased the HER2 immobilization propensity by a factor of ~4. Transient immobilization was even much more pronounced for the bipDARPin 6L1G that also showed higher oligomerization levels of 4–10 copies of HER2 as estimated from dual-color single-molecule co-localization analysis. These oligomer sizes are well in line with the expected daisy chain formation as depicted in Fig. 1d. The substantially higher oligomerization levels as compared to the 3–4 HER2 copies identified in chemically crosslinked oligomers could be explained by the limited crosslinking efficiency manifested by crosslinking of mainly DARPin-dimerized HER2.

**Fig. 8:**
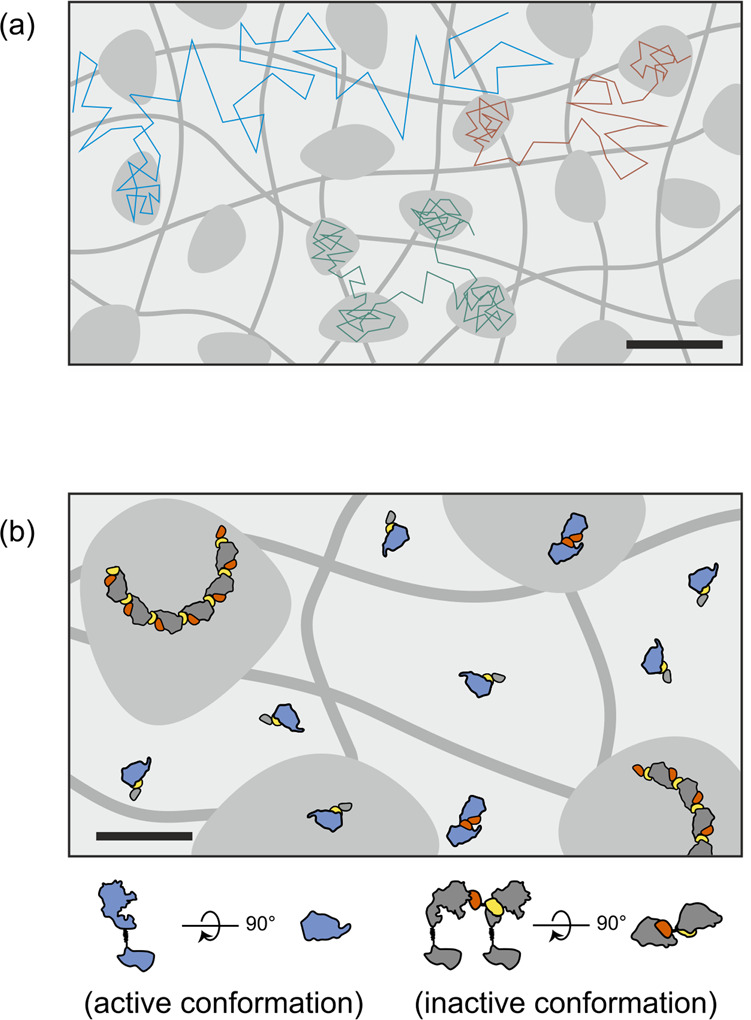
Putative mechanisms of HER2 lockdown and inhibition in the plasma membrane. **a** Characteristic diffusion modes of HER2 monomers (cyan), dimers (red), and oligomers (green) induced by different agents are due to differential propensity of partitioning into membrane nanodomains. Schematic trajectories and transiently forming nanodomains integrated over time. Trajectory time resolution: ~5 ms; hop diffusion inflicted by the membrane skeleton omitted for clarity. Scale bar: 50 nm. **b** Efficient inhibition of HER2 by bipDARPins caused by arrest in an inactive conformation upon formation of daisy chains and by strongly enhanced partitioning of HER2 daisy chains into membrane nanodomains. Scale bar: 20 nm.

Strikingly, 6L1G-cross-linked HER2 still showed residual mobility with a strongly increased propensity for transient arrest. In conjunction with the largely unaltered dwell time in the arrested state as compared to monomeric and dimeric states, these diffusion characteristics point to transient partitioning into membrane nanodomains rather than simply the oligomerization-induced decreased hopping probability across the picket fence of the cortical actin cytoskeleton (membrane skeleton (MSK)) as the fundamental basis of heterogeneous diffusion in the plasma membrane (Fig. 8a)^38–40^. Nanodomains in the mammalian plasma membrane have been related to cooperative interactions of lipids and proteins^36^. Thus, rather than partitioning, HER2 dimerization and oligomerization could increase the propensity of nanodomain formation. Our observations are in line with the concept of a hierarchical organization of the plasma membrane, with lipids and proteins transiently segregating into nanodomains within the framework of the MSK^34,41^.

While the molecular basis responsible for HER2-specific nanodomain partitioning/formation remains speculative, the intrinsic propensity of HER2 for transient arrest in the absence of crosslinking agents is evident. The drastic increase in partitioning propensity with increasing oligomerization level is in line with the cooperativity required for nanodomain formation and partitioning^41^. Based on MSD analysis, we estimate the size of the nanodomains to be below 50 nm, with residual mobility within the nanodomain (or even of the entire nanodomain). These zones do not seem to be related to endocytosis, as no increased endocytosis has been observed for bipDARPin-treated HER2^10^. Furthermore, partitioning and transient arrest, which are unaffected by extensive treatment with potent inhibitors of the HER2 kinase, do not appear to be related to signaling, as previously described for other receptors^37,42,43^.

On the contrary, our data implicate the possibility that transient arrest of HER2 obstructs downstream signaling, as we find a correlation between diffusional lockdown (i.e., uptake into nanodomains) and neutralization of signaling activity. However, mixtures of TZB and PZB (the approved combination for clinical therapy) or of homo-bivalent DARPins 6L46 and GL4G yielded strong lockdown of HER2 diffusion to a similar level as bipDARPins. Yet, the clinical benchmark combination of TZB and PZB neither potently neutralizes downstream signaling nor causes apoptosis in the absence of cellular cytotoxicity, as has been shown previously^10,44,45^. Therefore, lockdown is necessary but not sufficient for inducing apoptosis.

This highlights that the specific binding geometry of 6L1G is required for complete kinase inhibition^10,46^, via formation of signaling-inactive oligomers (Fig. 8b), which also preclude interaction with EGFR and HER3^10^. As we find that immobilization also occurs on cell lines resistant to HER2-targeted treatment^25^, diffusional arrest is a feature of a novel mechanism of action, in which loss of mobility precedes—and is a prerequisite for—signaling inhibition^10^. In therapeutic applications, it is attractive to increase plasma exposure either through conjugation to polymers to increase the molecular size above the renal filtration threshold or exploiting FcRn-mediated salvage pathways through albumin-binding domain or Fc region-containing molecules^47^. Of note, the mechanism of action described here is compatible with both approaches: PEGylation slightly affects the in vitro potency but not the efficacy of bipDARPins^10^, and the biparatopic targeting mechanism could be successfully transferred to IgG-based antibody constructs^48^. Taken together, these insights suggest that deliberate daisy chaining of HER2 potently downregulates its activity by a combination of conformational and diffusional arrest and thus may represent a generic strategy to achieve pan-receptor inhibition.

## Methods

### Protein production and labeling

DARPin constructs for treatment were expressed and purified as previously described^11,13^. PZB (Perjeta®) was a kind gift from Professor Uwe Zangemeister-Wittke and TZB (Herceptin®) was obtained from Kantonsapotheke Zürich. Affibody Zher2 (ref. ^22^) containing a C-terminal cysteine was coupled to maleimide-containing dyes, either Oregon Green 488 (Thermo Fisher, cat. no. O6034) or DY-647P1 (Dyomics, cat. no. 647P1-03) as previously described^49^.

### Generation of inducible HT-HER2 cell line

Stable cell lines for inducible expression of the HT-HER2 fusion were generated using the FlpIn TREx system (Thermo Fisher, cat. no. K650001). First, the human HER2 cDNA (Mammalian Gene Collection, GenBank accession number BC156755.1) without the signal peptide was amplified by PCR. The amplicons were subcloned into a derivative of the pcDNA5/FRT/TO plasmid, which contains the following features: The *Bsa*I site in the *bla* gene was removed by a silent mutation; in the expression cassette, a consensus Kozak sequence containing the start codon is followed by a murine κ light chain signal peptide, followed by the HaloTag7 sequence^50^. The coding sequence of mature HER2 was inserted using *Not*I and *Xho*I downstream of the HaloTag7, connected through a short glycine-serine linker, yielding an N-terminally HaloTagged HER2 targeted to the cell surface.

Sub-confluent cultures of Flp-In T-REx 293 cells (Thermo Fisher, cat. no. R78007) were then co-transfected with the pcDNA5/FRT/TO-derived plasmid containing HaloTag-HER2 and the Flp recombinase vector pOG44 using TransIT-293 (Mirus Bio, cat. no. 2700), following instructions provided by the manufacturers. In all, 150 µg ml^−1^ hygromycin B (Invitrogen, cat. no. 10687-10) in complete medium was used to select for stably transfected clones. After isolation using cloning discs (Sigma-Aldrich, cat. no. Z374431), single clones were expanded and analyzed by flow cytometry for inducible expression of HaloTag-HER2 to obtain the HEK-TREx_HT-HER2 cell line.

### Crosslinking analysis

Semi-confluent BT474 cells were detached by Accutase treatment (Thermo Fisher, cat. no. AT104) for 5 min at 37 °C, washed with RPMI1640 medium containing 10% (v/v) fetal calf serum, and afterwards washed three times with 25 mM 4-(2-hydroxyethyl)-1-piperazineethanesulfonic acid (HEPES) at pH = 7.5 containing 150 mM NaCl. Cells were counted on a CASY cell counter (model “TT,” Omni Life Science), and 2 × 10^6^ cells per sample were transferred to 200 μl HEPES buffer containing 10 nM of each DARPin or IgG. Cells were incubated for 1 h at 37 °C. Afterwards, cells were placed on ice, washed two times with 1 ml HEPES buffer, and resuspended in 200 μl HEPES containing 2.5 mM bis[sulfosuccinimidyl]suberate (BS3) (Pierce, cat. no. 21580). Crosslinking was performed for 1 h at room temperature; afterwards the reaction was quenched by washing twice with 1 ml of a 50 mM tris(hydroxymethyl)aminomethane (Tris) buffer at pH = 7.5 containing 150 mM NaCl. Cells were lysed for 15 min in 50 mM Tris buffer containing 1% Triton X-100 and protease and phosphatase inhibitors at 4 °C. The following protease inhibitors were used at the indicated final concentrations: 1 mM pefabloc SC (4-(2-aminoethyl)-benzolsulfonylfluorid-hydrochloride, Merck, cat. no. 124839), 0.02 mM leupeptin (Serva, cat. no. 51867), 0.01 mM pepstatin A (Serva, cat. no. 52682), and 0.02 mM marimastat (Calbiochem, cat. no. 444289). The following phosphatase inhibitors were obtained from Sigma Aldrich and used at the indicated final concentrations: 1 mM sodium orthovanadate (cat. no. 220590), 1 mM sodium metavanadate (cat. no. 72060), 10 mM sodium molybdate (cat. no. 331058), 50 mM sodium β-glycerol phosphate (cat. no. G9422), and 50 mM sodium fluoride (cat. no. 71519).

Lysates were kept on ice, cleared by centrifugation for 10 min at 20,000 × *g* at 4 °C, and supernatants were prepared for sodium dodecyl sulfate (SDS)-polyacrylamide gel electrophoresis (PAGE) in non-reducing SDS sample buffer (Invitrogen) and boiled for 3 min. Samples were loaded on NuPAGE 4–12% gradient gels (Invitrogen) and afterwards blotted at 100 V for 75 min onto activated PVDF-FL membrane (Millipore, cat. no. IPFL00010) using NuPAGE transfer buffer (cat. no. NP00061, Invitrogen) containing 10% MeOH.

The following antibodies were used for western blot analysis: anti-ErbB2 from mouse (Ventana, clone 4B5, cat. no. 790–2991), anti-mouse from goat conjugated to IRDye800 (Rockland, cat. no. 610-732-124), anti-rabbit from goat conjugated to Alexa Fluor 680 (Invitrogen, cat. no. A21076), anti-GAPDH from mouse (Santa Cruz, clone 6C5, cat. no. sc-32233), anti-ErbB2 from rabbit mAb (Santa Cruz, clone C-18, cat. no. sc-284), and anti-human IgG (heavy and light chain) from goat (Jackson ImmunoResearch). The anti-DARPin polyclonal serum was obtained by rabbit immunization (Dreier et al., unpublished). TZB (Herceptin®) and PZB (Perjeta®) for crosslinking experiments were obtained from Kantonsapotheke Zürich.

### HER2 labeling and treatments for FRAP

HEK-TREx_HT-HER2 cells were seeded at a density of 4 × 10^4^ cm^−2^ in Dulbecco’s modified Eagle’s medium (DMEM) without phenol red (Sigma-Aldrich, cat. no. D1145) supplemented with 10% (v/v) “tetracycline-free” fetal calf serum (Bioconcept, cat. no. 2-01F28-I), 4 mM L-glutamine (Sigma-Aldrich, cat. no. G7513), 1 mM sodium pyruvate (Sigma-Aldrich, cat. no. S8636), 1% (v/v) penicillin–streptomycin solution (Sigma-Aldrich, cat. no. P0781), and 150 µg ml^−1^ hygromycin B in µ-slides pre-coated with poly-L-lysine (Ibidi, cat. no. 80824) 2 days before a FRAP experiment. One day before the measurement, expression was induced by addition of a doxycycline (Applichem, cat. no. A2951) solution to a final concentration of 1 µg ml^−1^. Directly prior to FRAP data acquisition, HT-HER2 was labeled by addition of 400 nM HTL-AF660 (Promega, cat. no. G8472) and incubation for 15 min at 37 °C. Afterwards, the medium was removed, and the cells were carefully washed twice with Dulbecco’s phosphate-buffered saline (PBS) supplemented with 50 mM sodium azide (Sigma-Aldrich, cat. no. 71290) and 10 mM 2-deoxy-d-glucose (Sigma-Aldrich, cat. no. D8375) to yield PBSA50D. Cells were then treated by addition of the respective agent diluted to 100 nM in 200 µl PBSA50D, unless noted otherwise.

BT474 cells (ATCC) were seeded at a density of 10^5^ cm^−2^ in RPMI 1640 medium supplemented with 10% (v/v) fetal calf serum and 1% (v/v) penicillin–streptomycin in µ-slides with glass bottom (Ibidi, cat. no. 80827) 1 day prior to the experiment. For FRAP experiments, BT-474 cells were inactivated for 10 min in 100 µl PBSA50D, then labeled by adding 200 µl solution of Zher2-OG488 or Zher2-DY-647P1 (prepared as described previously^49^) in PBSA50D to yield a final concentration of 100 nM. Afterwards, cells were ATP-depleted by washing twice carefully with 300 µl PBSA50D, and 250 µl (125 µl each for sequential treatments) solution of the respective binding agent was added.

For the time-dependent observation of immediate immobilization, cells were labeled as above and measured before and repeatedly directly after addition of bipDARPin 6L1G.

For the observation of the long-term effects of treatment, 6L1G was added to the cells in complete medium and incubated for 5 h or 5 min, while labeling the cells at the same time by adding Zher2-DY-647P1 30 min before the wash step. Cells were then washed with PBSA50D as above.

For specific kinase inhibition, cells were pre-treated in complete medium for ~3 h with 12.5 µM HER2-specific kinase inhibitor (Selleckchem, cat. no. S2752, an analog of ARRY-380) or dimethyl sulfoxide. Afterwards, 6L1G was added to the respective samples and incubated for 10 min. Zher2-DY-647P1 was then added to all samples and allowed to bind for 30 min, and finally the cells were ATP-depleted by washing three times with PBSA50D before the measurement.

### Fluorescence recovery after photobleaching

Experiments were performed at room temperature, unless noted otherwise. We bleached a circular spot with 2 µm diameter, typically located in the equatorial plane^19^. Acquisition and bleach settings were optimized such that bleaching during acquisition was minimized (typically <5% of the initial fluorescence intensity was lost during the whole acquisition as determined by pre-bleach measurements) and to enable bleaching in a single step. If, for a single cell, <40% of initial fluorescence intensity was bleached within one single step (e.g., due to low level of labeled receptor for this cell, or a bleach spot not precisely centered on the membrane, or drift), this cell was excluded from analysis. Because cell death can be accompanied by a dramatic loss of lateral HER2 mobility (Supplementary Fig. 10), we confirmed at the end of all measurement periods (within ~1 h) that the mobility of untreated cells was essentially unaltered.

Image data including metadata were extracted from the proprietary Leica image file format (.lif) container, as put out by the instrument, with Fiji^51^ using the BioFormats importer^52^. We used a custom ImageJ script (available from the authors), which extracts the bleach region of interest (ROI) coordinates from the metadata, then allows manual positioning of a control ROI at a distant spot on the same cell (to correct for acquisition photobleaching), and exports the mean intensity values of bleach and control ROI. Data were scaled on a per-cell basis according to (*F*(*t*) − *F*(0)) ÷ (<*F*_pre_> − F(0)), where *F*(*t*) is the mean fluorescence in the ROI, with *t* = 0 defined at the first frame after the bleach frame, and <*F*_pre_> is the (spatial and temporal) mean fluorescence before the bleach step. Assuming a constant offset, the low background fluorescence cancels out in the linear transformation applied for normalization^53^. Afterwards, we corrected for the minor bleaching remaining under optimized settings by averaging single exponential fits to the mean fluorescence intensity of the control ROI^53^. Unless noted otherwise, plotted lines indicate mean values of at least five cells per condition.

### Single-molecule localization microscopy

For single-molecule localization experiments, an N-terminal fusion of the SNAP_f_ tag to HER2 (SNAP-HER2), constructed analogously to the HT-HER2 fusion (see above), was used for transient expression in HeLa cells. To this end, HeLa cells were seeded in dishes (6 cm diameter). On the next day, 2.5 µg of plasmid in a volume of 2.5 µl was mixed with CaCl_2_ in sterile water to yield a final concentration of 250 mM CaCl_2_ in a total volume of 500 µl. The resulting solution was slowly added to 500 µl of 2× HBSS solution (280 mM NaCl, 50 mM HEPES, 1.5 mM Na_2_HPO_4_, pH = 6.95–7.05, sterile-filtered) with agitation and incubated for 10 min at room temperature. Afterwards, the suspension was evenly added to the cell dish, and the cells cultivated for ~7 h at 37 °C in a humidified 3% CO_2_ atmosphere, then detached and plated overnight onto No. 1.5 coverslips that were pre-treated with a 1 mg ml^−1^ PLL-PEG-RGD solution, which was synthesized as described previously^54^, to minimize non-specific binding of dye molecules to the coverslip surface^28^.

SNAP_f_-HER2 was labeled by incubation with a mixture of 80 nM SNAP-Surface 649 (DY-649P1, NEB) and 8 nM SNAP-Surface 549 (DY-549P1, NEB) in 1 ml phenol red-free medium (DMEM) for 10 min at 37 °C, and the cells were afterwards washed twice with Dulbecco’s PBS. These concentrations of SNAP substrates ensured similar labeling efficiencies in both channels. All imaging experiments were carried out at room temperature (to minimize endocytosis of HER2) in phenol red-free medium (DMEM) supplemented with an oxygen scavenger and a redox-active photoprotectant (0.5 mg/ml glucose oxidase (Sigma-Aldrich), 0.04 mg/ml catalase (Roche), 5% w/v glucose, 1 μM ascorbic acid, and 1 μM methyl viologen) to reduce blinking and photobleaching of the fluorophores^55^. Treatments (DARPins and antibodies) were added to a final concentration of 100 nM.

Dual-color single-molecule imaging was performed using TIRFM with an inverted microscope (Olympus IX71) equipped with a triple-line total internal reflection illumination condenser (Olympus) and a back-illuminated electron multiplying charge-coupled device (EMCCD) camera (iXon DU897D, 512 × 512 pixels, Andor Technology) operated at −80 °C. A 150× magnification objective with a numerical aperture of 1.45 (UAPO ×150/1.45 TIRFM, Olympus) was employed for sample illumination and fluorescence collection. For dual-color acquisition, DY-549P1 was excited by a 561 nm diode-pumped solid-state laser (CL-561-200; CrystaLaser) at 0.95 mW and DY-649P1 was excited by a 642 nm laser diode (LuxX 642-140, Omicron) at 0.65 mW (power output after passage of the objective). Fluorescence was detected using a spectral image splitter (DualView; Optical Insight) with a 640 DCXR dichroic beam splitter (Chroma Technology Corporation) in combination with a 585/40 band-pass filter (Semrock) for detection of DY-549P1 and a 690/70 band-pass filter (Chroma Technology Corp) for detection of DY-649P1, projecting the respective spectral channels on the upper and lower half of the EMCCD chip. Time series of 2000 images were recorded with a time resolution of 32 ms per frame.

### Single-molecule analyses

Single-molecule localization was achieved using the multiple-target tracing algorithm (MTT)^56^. Briefly, individual signals were first identified with a fixed false-positive rate of 10^−6^ using pixel-wise hypothesis-testing against the local background noise (9 × 9 pixel evaluation box) and then localized with sub-pixel precision (typically 20 nm) approximating the microscope’s point-spread function with a two-dimensional Gaussian profile with predetermined fixed radius. Spatiotemporal super-resolution images were rendered by placing all localization within the first 500 frames on an upsampled pixel grid blurred with the average localization precision as described in detail previously^57^.

Diffusion properties were determined only from the DY-649 channel because particle densities were more homogeneous. Prior to tracking, the data set was scanned for transient immobilization events (>640 ms or 20 frames) within a 120 nm search radius around each localization using an adapted version of the density-based spatial clustering of applications with noise principle^58^ as described earlier^59^. Individual positions of mobile particles were subsequently connected into trajectories (allowing for a three-frame-long observation gap) based on their locally characteristic displacement statistics calculated along each molecule trajectory^60^. MSDs of the tracked particles (>320 ms or 10 frames) were fitted according to MSD = 4*Dτ* − 4/3*D*Δ*t* + 4*ε*^2^, where *τ* is the time lag (duration of displacement), Δ*t* is the camera exposure time, and *ε* is the localization precision^61^, weighted by their expected inverse variances in order to obtain the instantaneous diffusion coefficient *D* (*τ* < 160 ms or 5 frames). Crosslinked receptors were identified by dual-color single-molecule co-tracking as described in detail previously^62^. Briefly, spectral channels were first aligned spatially with sub-pixel accuracy based on a prior calibration measurement with multicolor fluorescent beads (TetraSpeck microspheres, 100 nm, Invitrogen). The fraction of mobile points was subsequently probed for co-locomotion (consecutive co-localization within 150 nm for at least 320 ms or 10 frames). Fractions (immobile as well as dual-labeled) are calculated for each frame respective to the observed number of total localizations and reported as their average over time.

Alternation between mobile and immobile periods within individual trajectories was quantified by confinement analysis, as described previously^63^. Confinement was probed with a time resolution of 320 ms (10 frames) along individual trajectories (>3.2 s or 100 frames) against unrestricted motion of the HER2 monomer with an expected diffusion coefficient of 0.19 µm^2^ s^−1^ (OL4G). A controlled threshold to discriminate confined versus unhindered periods of diffusion was established based on simulation of free Brownian motion with a fixed false positive rate of 10^−3^. Finally, maximum-likelihood estimates of the transition probabilities were calculated from the obtained state sequence (https://www.cs.ubc.ca/~murphyk/Software/HMM/hmm.html).

In all box plots showing data derived from single-molecule localization and tracking experiments, the center line indicates the median, the box limits indicate upper and lower quartiles, the whiskers show the 1.5× interquartile range.

### Reporting summary

Further information on research design is available in the Nature Research Reporting Summary linked to this article.

## Supplementary information

Description of Additional Supplementary Files

Supplementary Data 1

Supplementary Data 2

Reporting Summary

Supplementary Movie 1

Supplementary Movie 2

Supplementary Movie 3

Supplementary Movie 4

Supplementary Movie 5

Supplementary Movie 6

Supplementary Movie 7

Supplementary Information

## Data Availability

Source data sets underlying Figs. 2–7 are provided in Supplementary Data 1. The extensive raw data sets generated during the current study are available from the corresponding author on reasonable request. All derived data and analyses supporting the findings of this study are included in this manuscript and its supplementary information files.

## Code availability

Unless previously published and referenced in the text, all software is available from the authors upon reasonable request.
