# Supplementary material for: Apoptosis-inducing anti-HER2 agents operate through oligomerization-induced receptor immobilization
Source: Commun Biol. 2021 Jun 21;4:762. doi: 10.1038/s42003-021-02253-4 (PMC8217238; doi:10.1038/s42003-021-02253-4)
Supplement: Supplementary file 1 — Description of Additional Supplementary Files [file 42003_2021_2253_MOESM1_ESM.pdf]

Description of Additional Supplementary Files

Supplementary Data 1: Data underlying the plots in Fig. 3,4,5,6,7.

Supplementary Data 2: Image data for Fig. 2,3,6. For Figure 2, uncropped blots are provided. For Figure 3, the raw data files (.lif) and extracted single frames (.tiff) at time points as indicated in the figure are provided. For Figure 6, rendered super-resolution images as shown in the figure are provided in original resolution.

Supplementary Movie 1: Spatiotemporal dynamics of HER2 in the plasma membrane of HeLa cells after treatment with the monovalent DARPin OL4G. Left: Cumulation of localized particles. Center: Time-lapse localization (white circles) and identification of immobile molecules (colored circles). Right: tracking of mobile molecules. Scale bar: 2 µm.

Supplementary Movie 2: Spatiotemporal dynamics of HER2 in the plasma membrane of HeLa cells after treatment with the active, apoptosis-inducing bipDARPin 6L1G. Left: Cumulation of localized particles. Center: Time-lapse localization (white circles) and identification of immobile molecules (colored circles). Right: tracking of mobile molecules. Scale bar: 2 µm.

Supplementary Movie 3: Transient arrest of HER2 diffusion in the plasma membrane of cells treated with the monovalent control DARPin OL4G. Time-lapse localization (white circle) and identification of immobile molecules (colored circle). Scale bar: 2 µm.

Supplementary Movie 4: Transient arrest of HER2 diffusion in the plasma membrane of cells treated with the apoptosis-inducing bipDARPin 6L1G. Time-lapse localization (white circles) and identification of immobile molecules (colored circles). Scale bar: 2 µm.

Supplementary Movie 5: Highly localized diffusion in transient confinement zones observed for HER2 after treatment with OL4G.

Supplementary Movie 6: Highly localized diffusion in transient confinement zones observed for HER2 after treatment with GL4G.

Supplementary Movie 7: Highly localized diffusion in transient confinement zones observed for HER2 after treatment with 6L1G.
