# Supplementary figures and images for: Apoptosis-inducing anti-HER2 agents operate through oligomerization-induced receptor immobilization
Source: Commun Biol. 2021 Jun 21;4:762. doi: 10.1038/s42003-021-02253-4 (PMC8217238; doi:10.1038/s42003-021-02253-4)

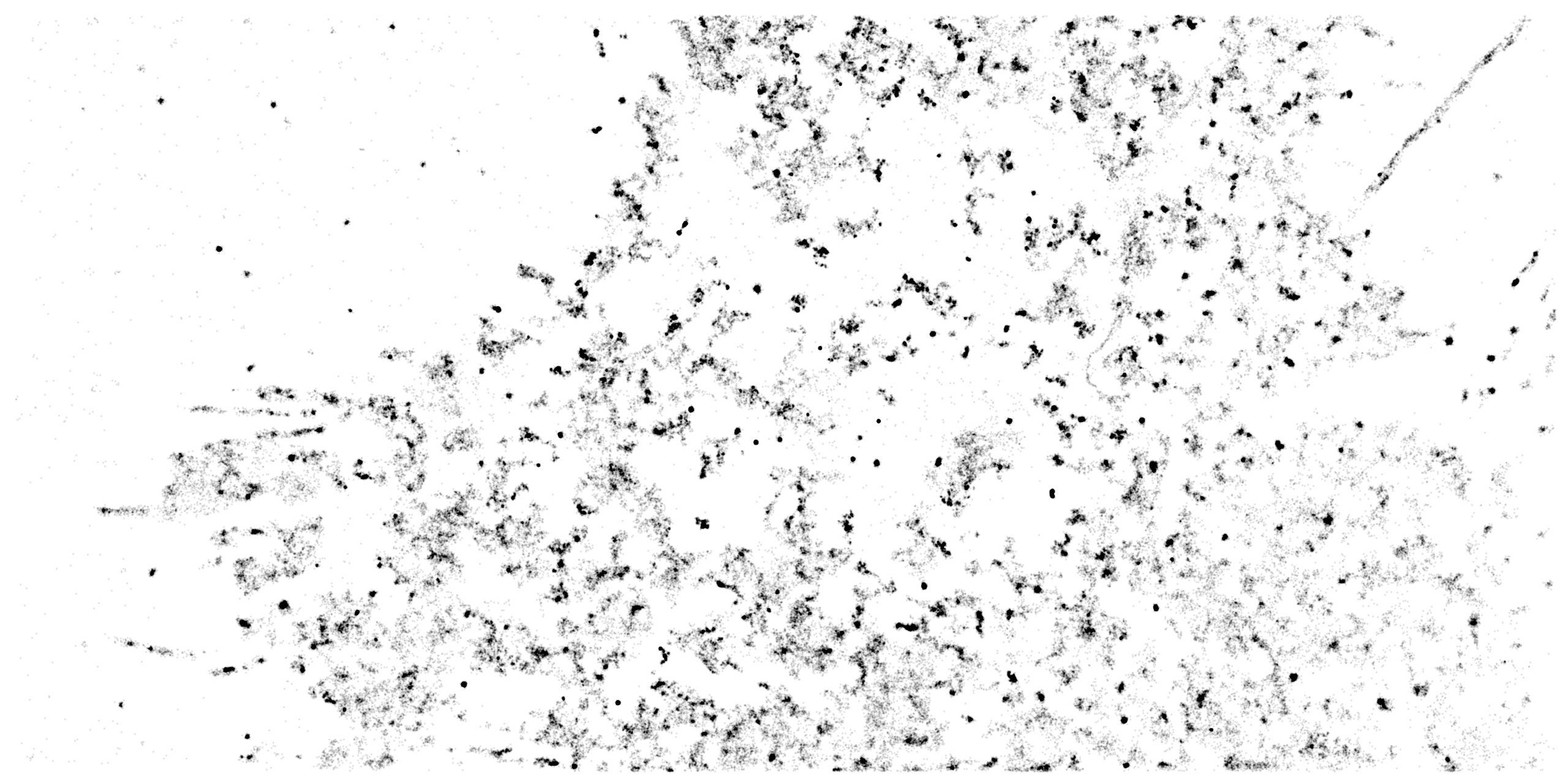

Supplement: Supplementary file 2 — Supplementary Data 1 [file 42003_2021_2253_MOESM2_ESM.zip › image_data/Figure-6/6-5-G-2.tif]

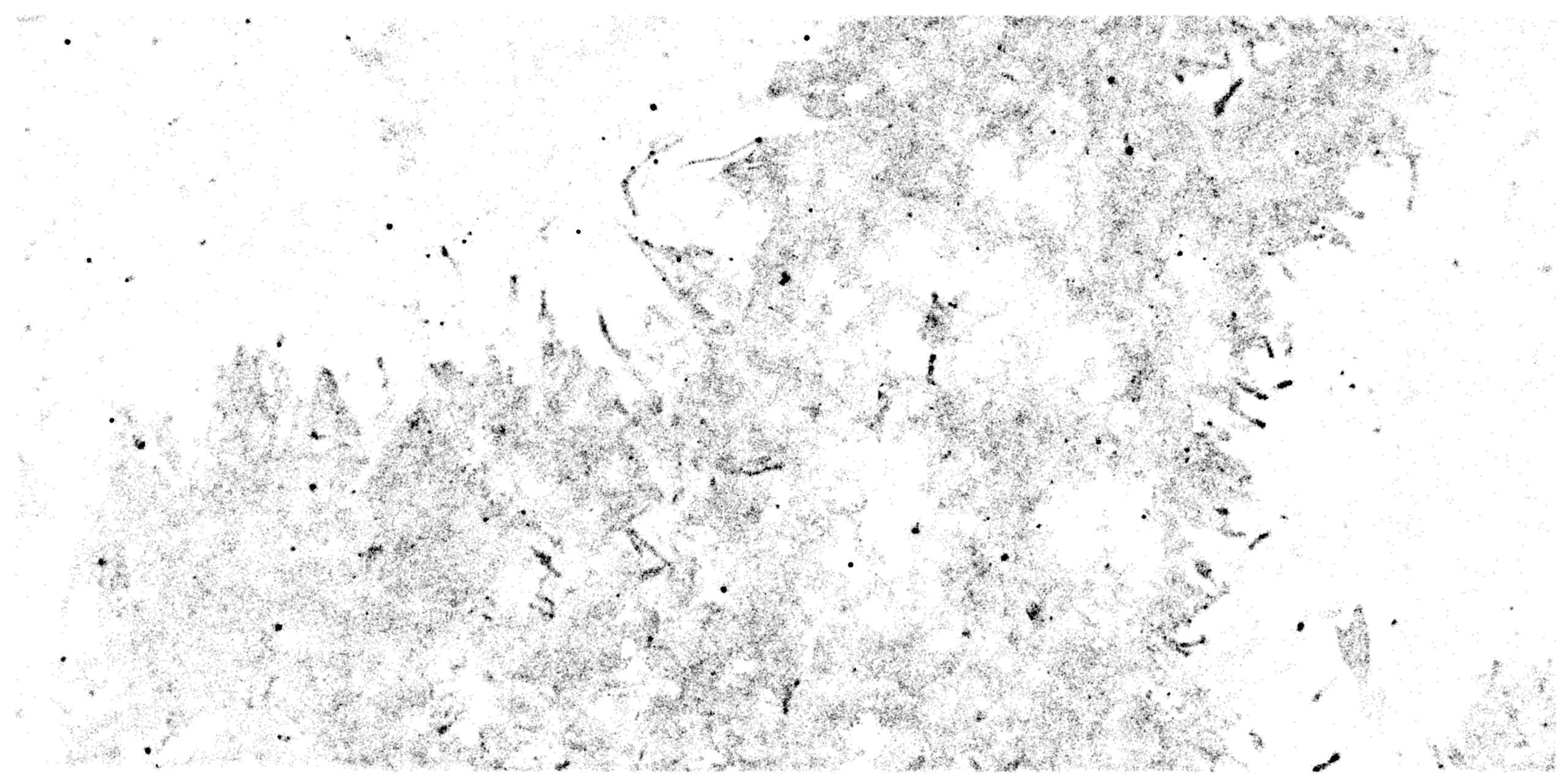

Supplement: Supplementary file 2 — Supplementary Data 1 [file 42003_2021_2253_MOESM2_ESM.zip › image_data/Figure-6/off7-3.tif]

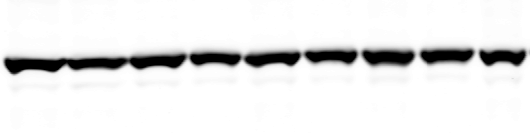

Supplement: Supplementary file 2 — Supplementary Data 1 [file 42003_2021_2253_MOESM2_ESM.zip › image_data/Figure-2/GAPDH_120501.png]

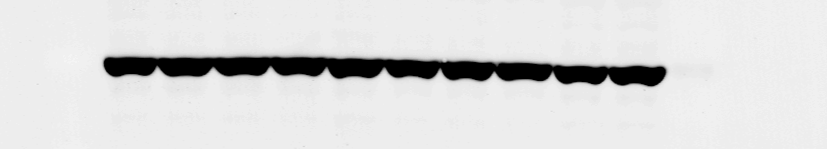

Supplement: Supplementary file 2 — Supplementary Data 1 [file 42003_2021_2253_MOESM2_ESM.zip › image_data/Figure-2/HER2_121223_GAPDH_CUT1.tif]

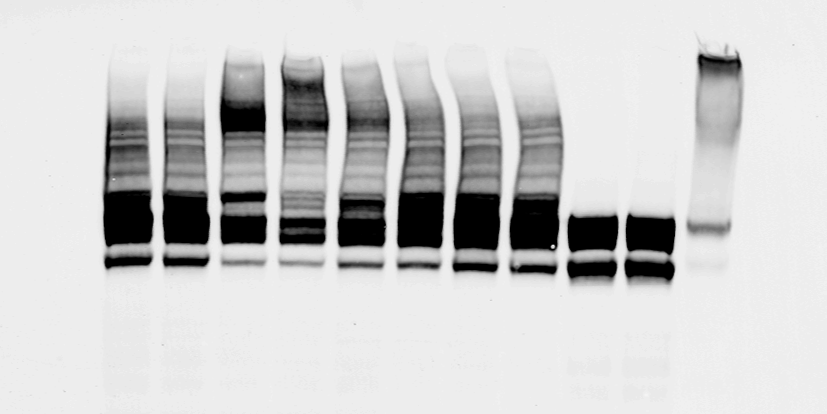

Supplement: Supplementary file 2 — Supplementary Data 1 [file 42003_2021_2253_MOESM2_ESM.zip › image_data/Figure-2/HER2_121223__HER2_CUT1.tif]

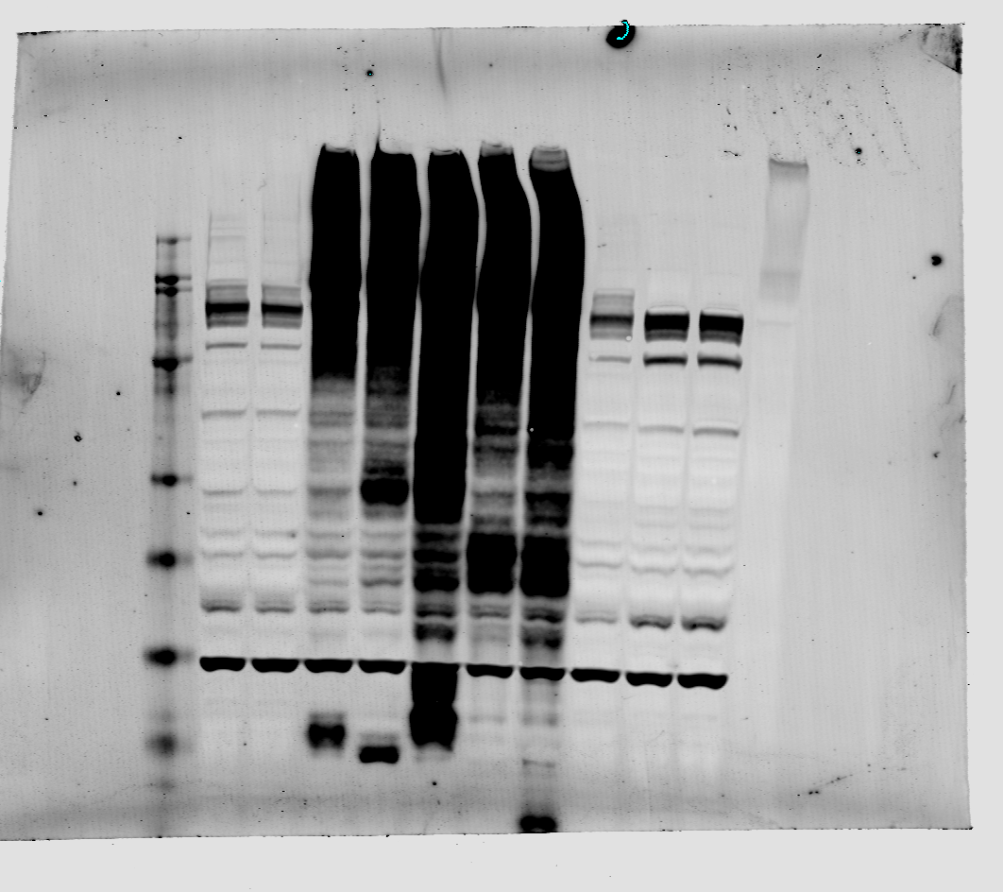

Supplement: Supplementary file 2 — Supplementary Data 1 [file 42003_2021_2253_MOESM2_ESM.zip › image_data/Figure-2/GAPDH_A_BNW.tif]

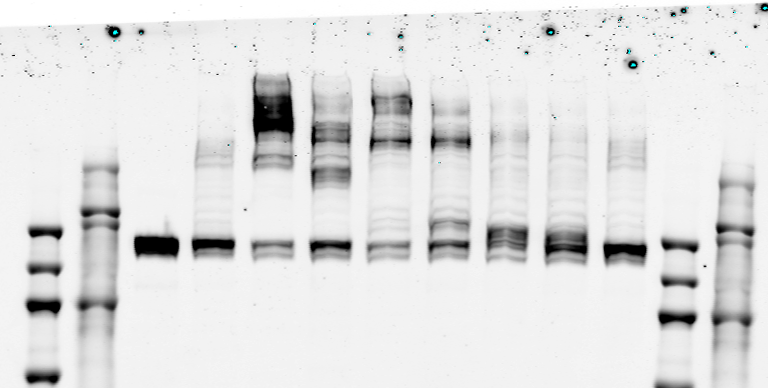

Supplement: Supplementary file 2 — Supplementary Data 1 [file 42003_2021_2253_MOESM2_ESM.zip › image_data/Figure-2/HER2_120501_B_HER2.tif]

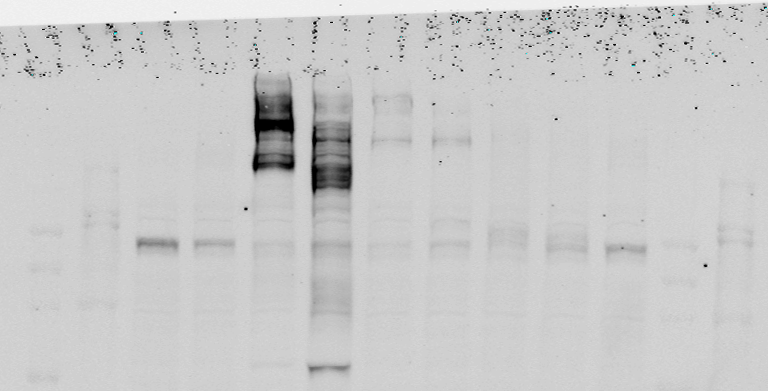

Supplement: Supplementary file 2 — Supplementary Data 1 [file 42003_2021_2253_MOESM2_ESM.zip › image_data/Figure-2/HER2_120501_B_IgG.tif]

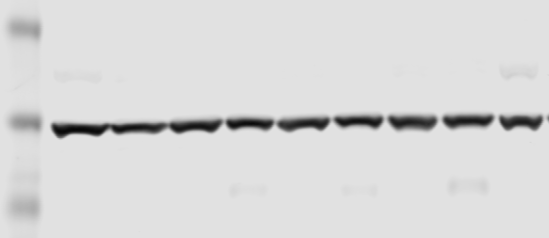

Supplement: Supplementary file 2 — Supplementary Data 1 [file 42003_2021_2253_MOESM2_ESM.zip › image_data/Figure-2/GAPDH_B_HER2_120501_D1_BNW.tif]

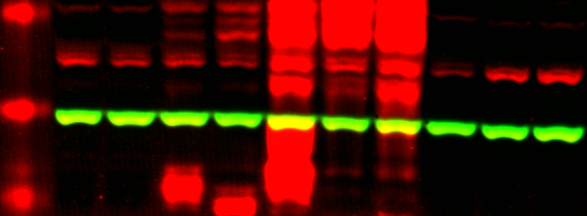

Supplement: Supplementary file 2 — Supplementary Data 1 [file 42003_2021_2253_MOESM2_ESM.zip › image_data/Figure-2/GAPDH_A_121223_Col.tif]

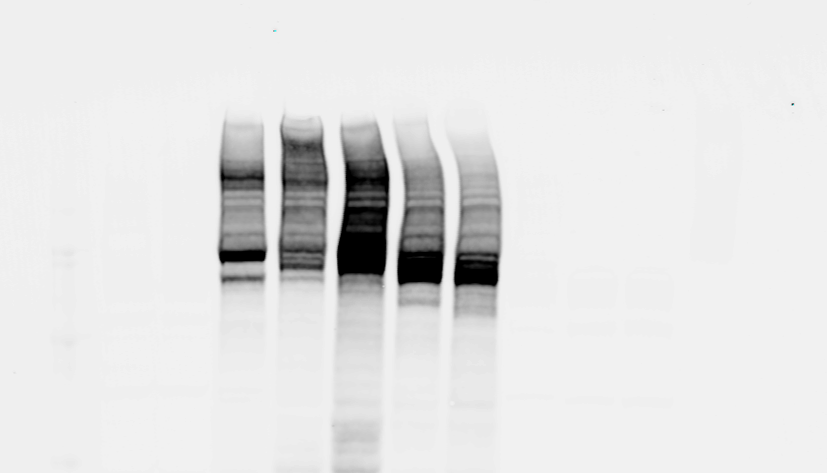

Supplement: Supplementary file 2 — Supplementary Data 1 [file 42003_2021_2253_MOESM2_ESM.zip › image_data/Figure-2/HER2_121223__DARPin_CUT1.tif]

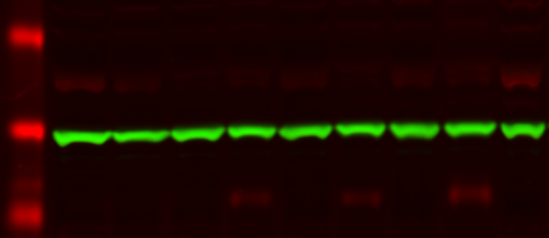

Supplement: Supplementary file 2 — Supplementary Data 1 [file 42003_2021_2253_MOESM2_ESM.zip › image_data/Figure-2/GAPDH_B_HER2_120501_D1.tif]
