# Supplementary material for: Apoptosis-inducing anti-HER2 agents operate through oligomerization-induced receptor immobilization
Source: Commun Biol. 2021 Jun 21;4:762. doi: 10.1038/s42003-021-02253-4 (PMC8217238; doi:10.1038/s42003-021-02253-4)
Supplement: Supplementary file 12 — Supplementary Information [file 42003_2021_2253_MOESM12_ESM.pdf]

Supplementary material

Apoptosis-inducing anti-HER2 agents operate through oligomerization-induced receptor immobilization

Jakob C. Stüber[1,3#], Christian P. Richter[2#], Junel Sotolongo Bellón[2], Martin Schwill[1], Iwo König[1,4], Benjamin Schuler[1], Jacob Piehler[2‡], & Andreas Plückthun[1‡]

[1]Department of Biochemistry, University of Zurich, Winterthurerstrasse 190, CH-8057 Zurich, Switzerland

[2]Department of Biology/Chemistry and Centre for Cellular Nanoanalytics, University of Osnabrück, Barbarastrasse 11, 49076 Osnabrück, Germany

[3]Present address: Roche Pharma Research & Early Development, Large Molecule Research, Roche Innovation Center Munich, Nonnenwald 2, 82377 Penzberg, Germany

[4]Present address: Roche Diagnostics Int. AG, Forrenstrasse 2, CH-6343 Rotkreuz, Switzerland

[‡]Corresponding authors, e-mail: piehler@uos.de or plueckthun@bioc.uzh.ch, Fax: +41-44-635-5712

Supplementary figures

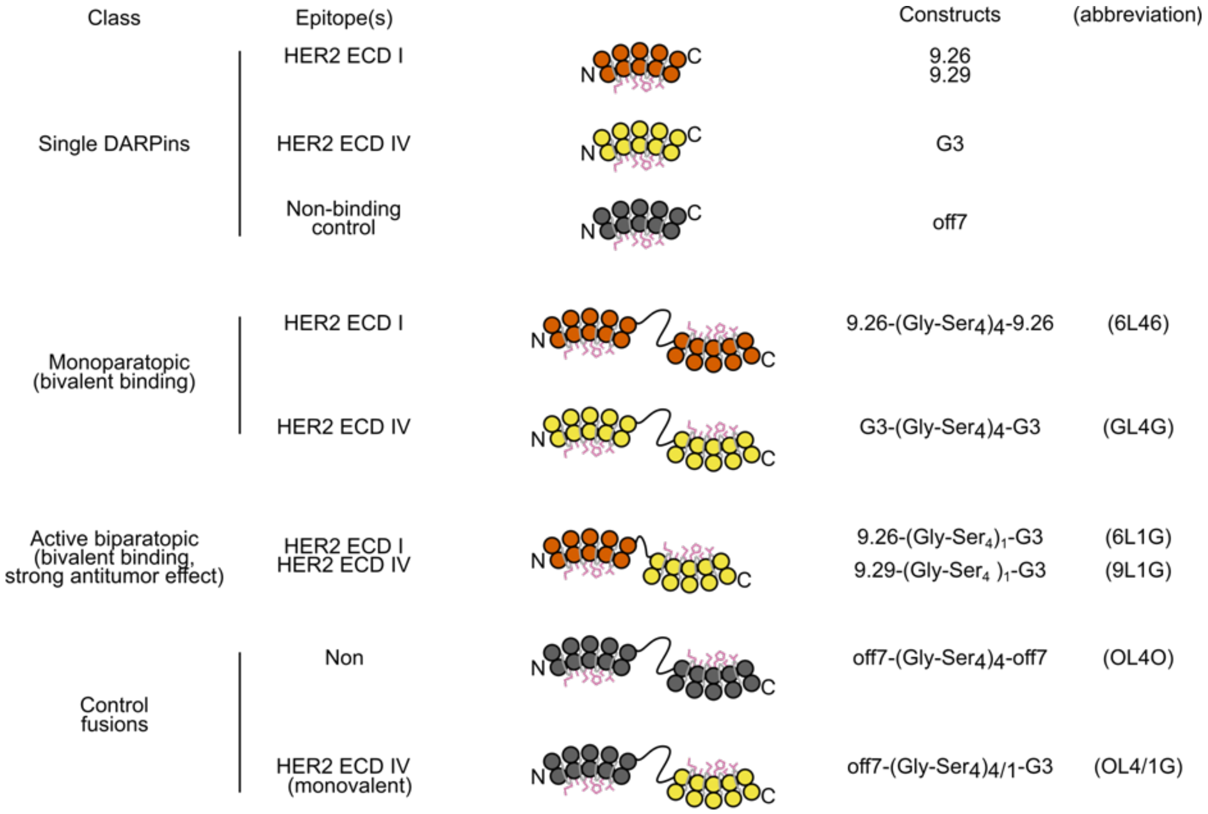

Supplementary Figure 1: Overview of DARPin constructs used in this study. Abbreviation: *ECD*, extracellular subdomain.

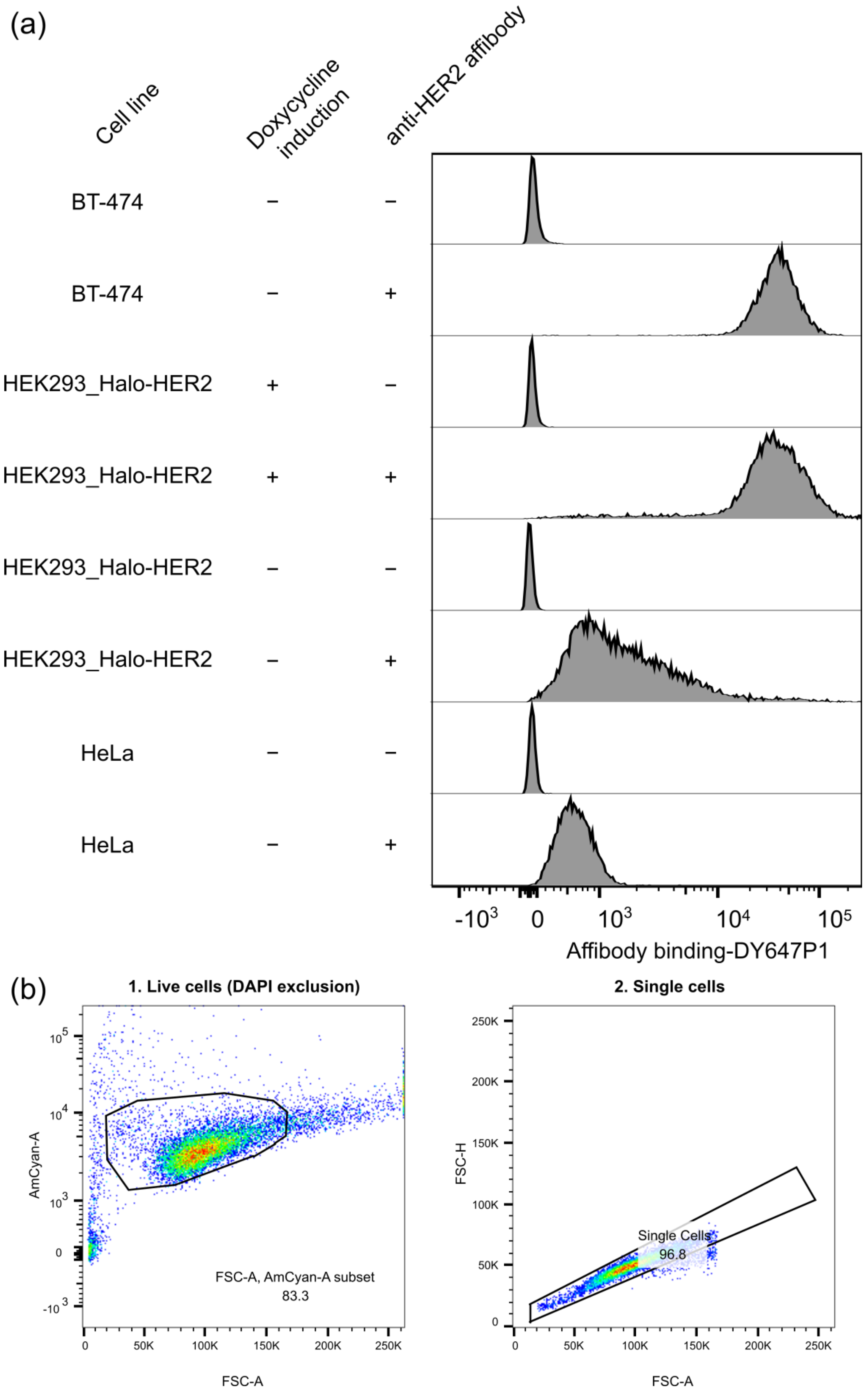

Supplementary Figure 2: (a) Histograms of the flow cytometry data used in generation of Supplementary Table 1. Total HER2 expression (endogenous native HER2 and HaloTag-HER2 fusion, where present) was detected using the affibody Z_{HER2} conjugated

to DY-647P1. For the HEK293_Halo-HER2 cell line, expression was induced by doxycycline addition where indicated. (b) Gating strategy applied in flow cytometry experiments shown in (a) and Supplementary Table 1. Intact cells were first identified as particles in the appropriate size range, which are inaccessible to a permeability marker (DAPI). A plot of the forward scatter peak area versus the peak height was then used to gate for single cells.

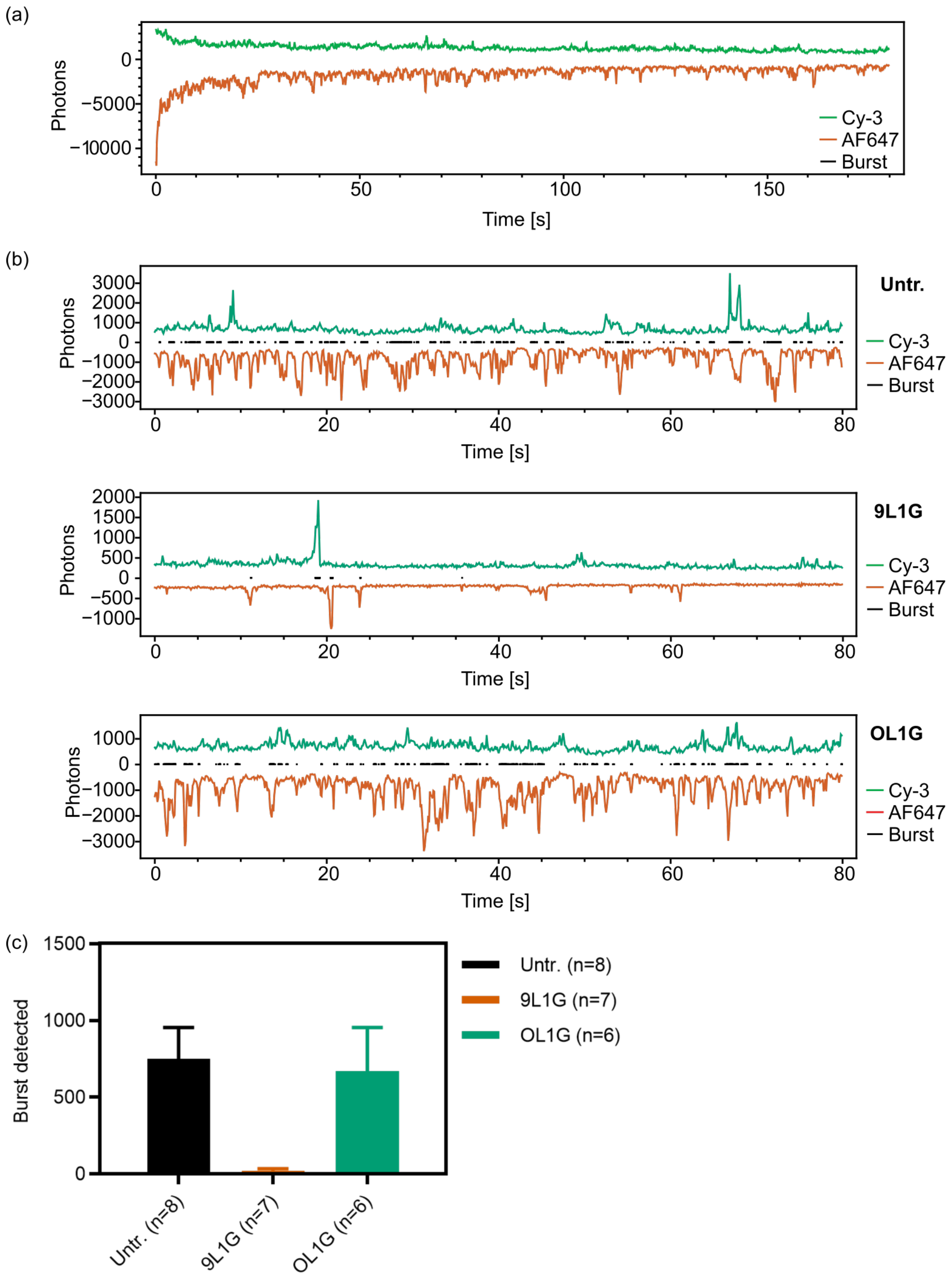

Supplementary Figure 3: Single-molecule confocal spectroscopy in a HEK293_Halo-HER2 cell line suggests that bipDARPins induce HER2 immobilization. (a) HT-HER2 was labeled with a mixture of HaloTag ligands carrying Alexa Fluor 647 (HTL-AF647) or Cy3 (HTL-Cy3). At 5 µW laser power, a pronounced initial bleaching phase was followed a by a steady state with distinct single-molecule bursts (binning: 100 ms). (b)

After addition of biologically active, apoptosis-inducing DARPin 9L1G, the burst count in the steady state is significantly reduced compared to non-treated cells or cells treated with monovalent control-DARPin OL1G (see Supplementary Figure 1), which can only bind a single HER2 molecule. The first 100 s, where the background signal is not constant due to bleaching, were excluded from burst detection (binning: 100 ms for all samples). (c) Results of automated burst counting after treatments as in (b). Error bars represent the SEM.

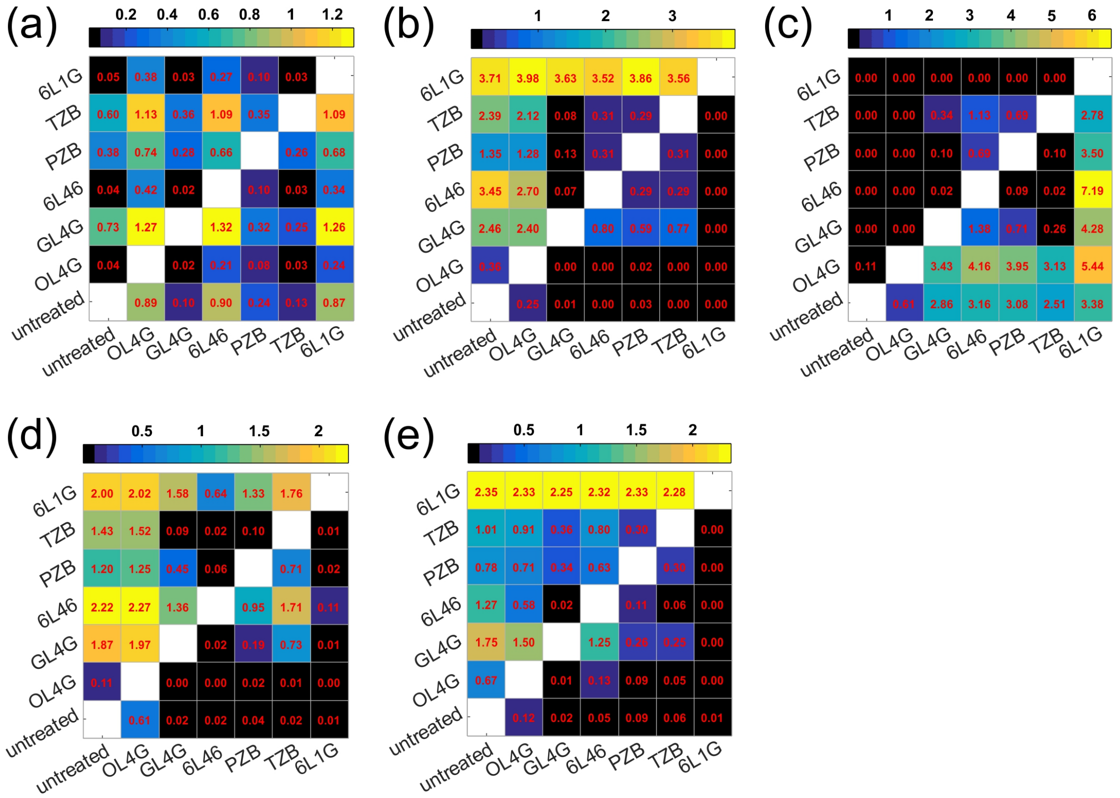

Supplementary Figure 4: Statistical significance for data shown in Fig. 6b-f. Pair-wise significance testing (Welch one-sided t-test with unequal sample size and variance) of localization density of HER2-fSNAP labelled with DY-649 (a), immobile fraction (b), diffusion coefficient of the mobile phase estimated by mean squared displacement (MSD) analysis (c), fraction of points being both mobile and co-localized in both spectral channels (d) and fraction of points being both immobile and co-localized in both spectral channels (e). The significance matrix is read left-to-right color-coded by the reduction risk (RR = –lg(p-Value): ns, RR < 1.3; *, RR ≥ 1.3; **, RR ≥ 2 and ***, RR > 3).

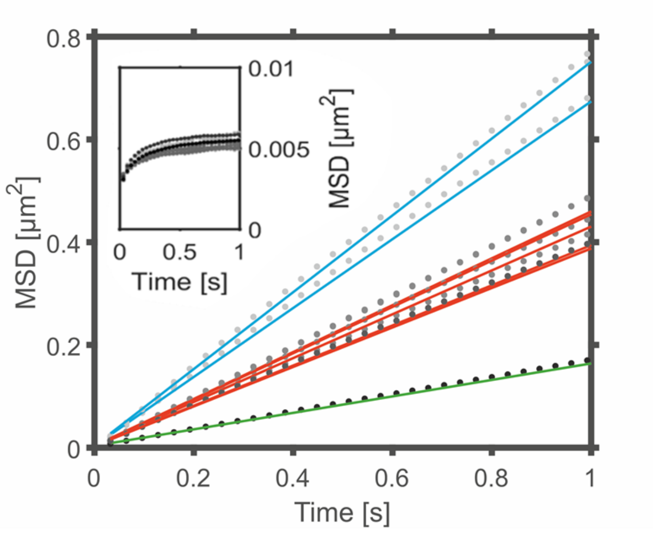

Supplementary Figure 5. Diffusion properties of HER2 evaluated by mean squared displacement (MSD) analysis. Pooled MSDs of the mobile periods (and the immobile periods in the inset) from single-molecule trajectories of HER2 treated with different agents. Different colors indicate the three distinct groups of agents (cf. Figure 6) as referenced in the main text.

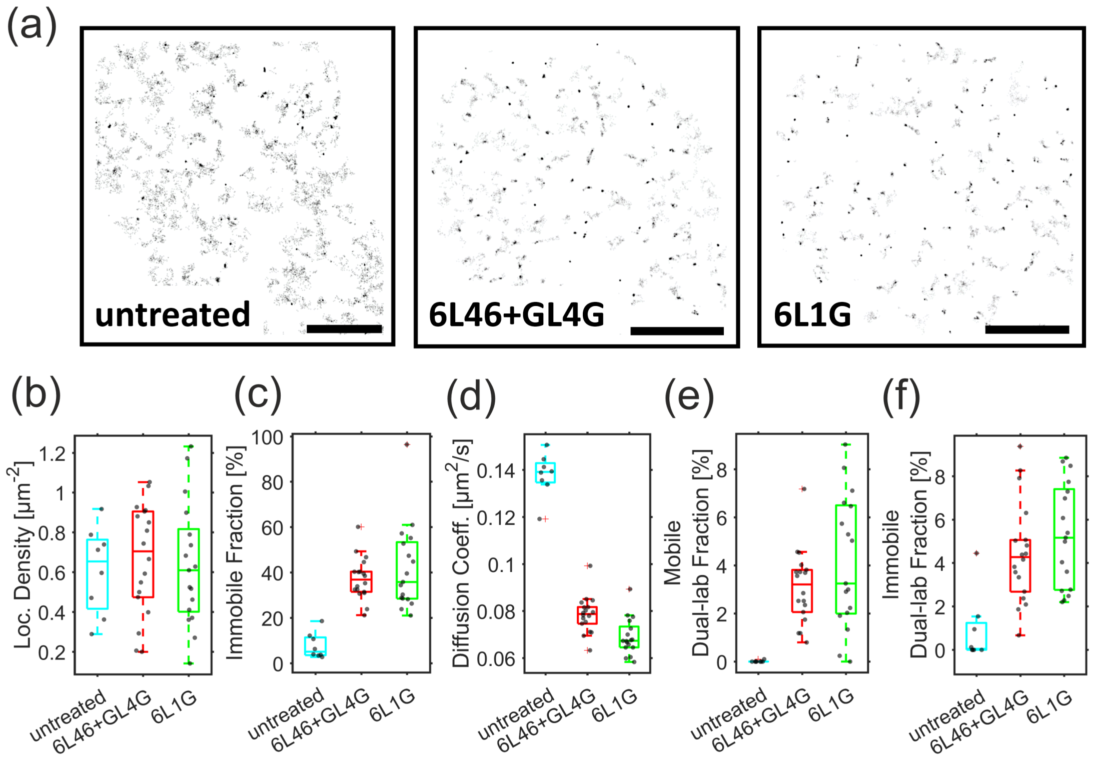

Supplementary Figure 6. Diffusional arrest by 6L1G is equivalent to crosslinking by the combination of monovalent but biparatopic 6L46 and GL4G. (a) Representative SMLM super-resolution images rendered from 150 consecutive frames with localization densities encoded as grey values. Scale bar: 5 µm. (b) Particle densities in the DY-649 channel observed in different single-molecule experiments. (c, d) Comparison of the immobile fraction identified by spatiotemporal clustering analysis (c) and the diffusion coefficient within the mobile fraction (d) for HER2 for different treatments. (e, f) Colocalized HER2 molecules in the mobile (e) and the immobile fraction (f). The DARPins are shown schematically in Supplementary Figure 1. Significance values for panels b-g are provided in Supplementary Figure 7.

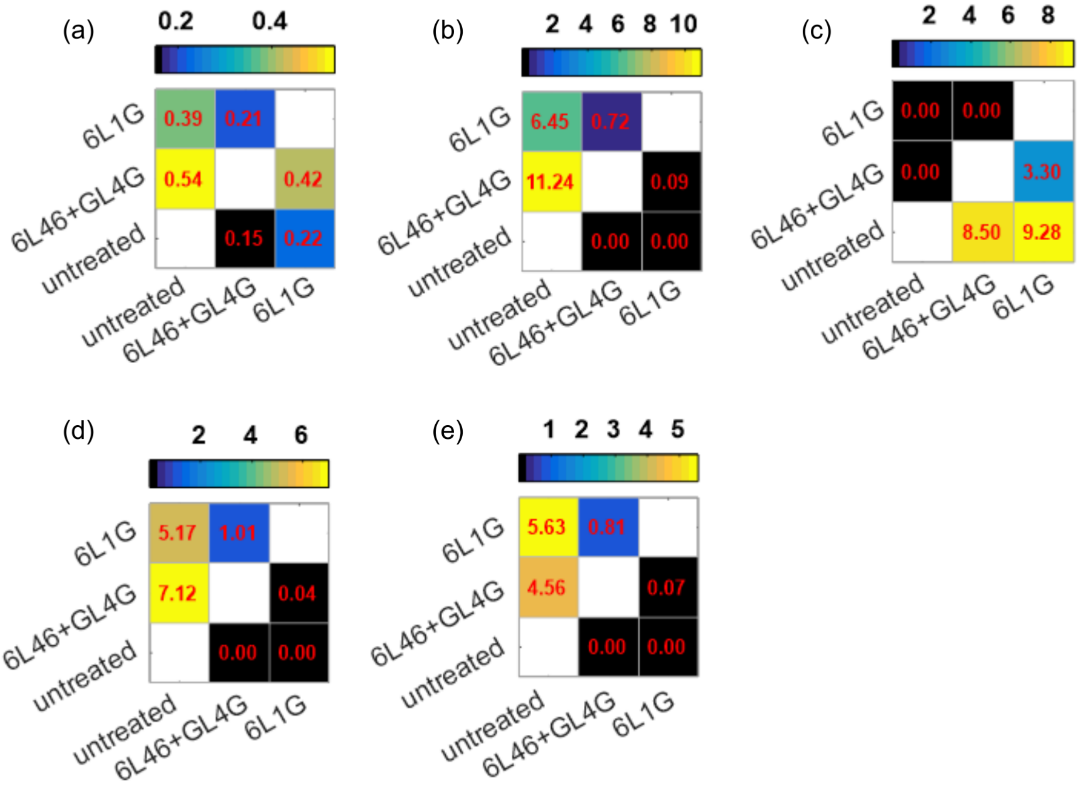

Supplementary Figure 7. Statistical significance for data shown in Supplementary Figure 6b-f. Pair-wise significance testing (Welch one-sided t-test with unequal sample size and variance) of localization density of HER2-fSNAP labelled with DY-649 (a), immobile fraction (b), diffusion coefficient of the mobile phase estimated by mean squared displacement (MSD) analysis (c), fraction of points being both mobile and co-localized in both spectral channels (d) and fraction of points being both immobile and co-localized in both spectral channels (e). The significance matrix is read left-to-right color-coded by the reduction risk (RR = –lg(p-Value): ns, RR < 1.3; *, RR ≥ 1.3; **, RR ≥ 2 and ***, RR > 3).

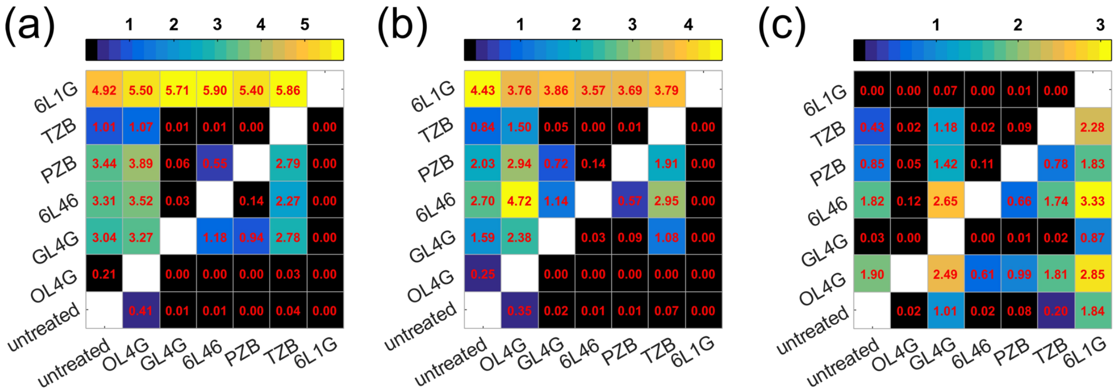

Supplementary Figure 8: Statistical significance for data shown in Fig. 7d-f. Pair-wise significance testing (Welch one-sided t-test with unequal sample size and variance) for the (a) Identified confined fraction (b) the transitioning probability into confined motion and (c) the reverse transition probability out of confined into free motion. The significance matrix is read left-to-right color-coded by the reduction risk (RR = –lg(p-Value): ns, RR < 1.3; *, RR ≥ 1.3; **, RR ≥ 2 and ***, RR > 3).

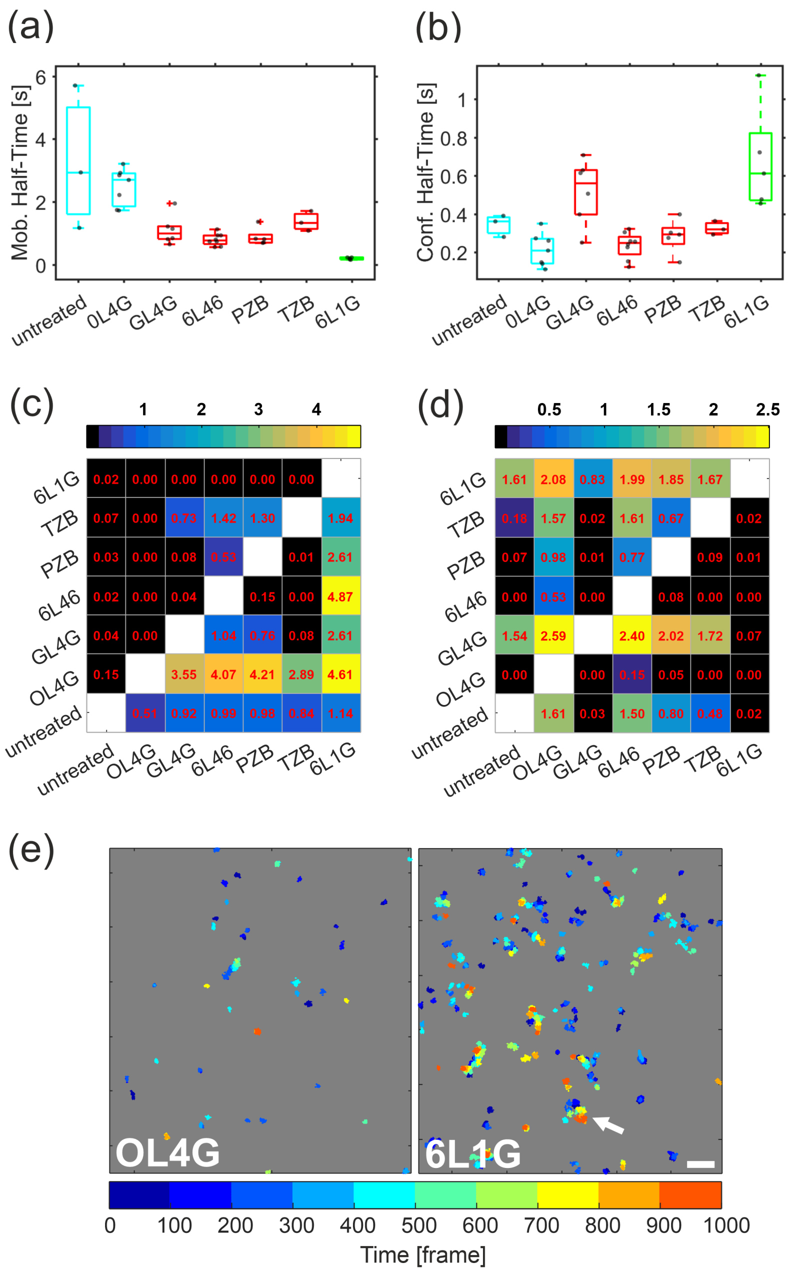

Supplementary Figure 9. Spatiotemporal dynamics of transient arrest of HER2 diffusion. (a, b) Half-lives calculated from the estimated probabilities for transitioning from free to arrested diffusion (a) and, vice versa, transitioning from confined to free motion (b), as obtained from confinement analysis. (c, d) Respective pair-wise significance testing (Welch one-sided t-test with unequal sample size and variance) for the mobile half-life (c) and the arrested half-life (d). The significance matrix is read left-to-right color-coded by the reduction risk (RR = –lg(p-Value): ns, RR < 1.3; *, RR ≥ 1.3; **, RR ≥ 2 and ***, RR > 3). (e) Detected immobilization events color-coded by time for

monovalent OL4G and biparatopic 6L1G. The white arrow points at immobilization events scattered across space over time as detected by spatiotemporal clustering. Temporal color-coding of the trajectories according to the legend in the bottom (in milliseconds). Scale bar: 1 µm. The overall particle density was very similar in both experiments.

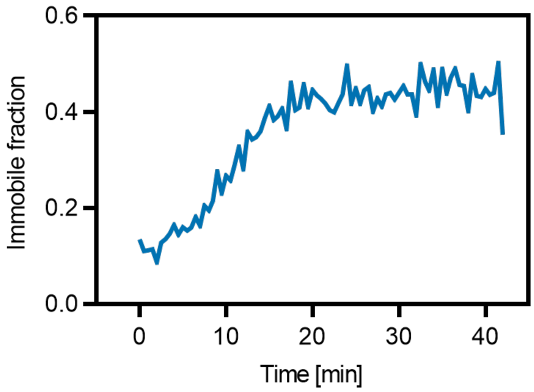

Supplementary Figure 10: Metabolic inhibition (ATP depletion) is unsuitable for single-molecule tracking experiments because it leads to rapid loss of receptor mobility if combined with photoprotection cocktails. Cells were exposed to a photoprotection cocktail[1] combined with metabolic inhibitors NaN_3 and 2-deoxy-D-glucose in PBS to achieve ATP depletion[2]. The time course of immobilization, measuring 30 frames of 0.020 s every 30 s, was quantified using the DBSCAN algorithm[3].

Supplementary tables

Supplementary Table 1: Estimation of total HER2 expression (HaloTag fusion and wt HER2) on various cell lines.

Cell line[1]	Rel. mean surface expression			Estimated[2] mean number of receptors per cell			Estimated[3] mean cell surface density [μm^{-2}]		
Detection with anti-HER2 affibody[4]									
BT-474	100.0%			870,000			693		
HEK293_Halo-HER2, induced	100.8%			877,297			698		
HEK293_Halo-HER2, non-induced	8.1%	±	0.9%	70,323	±	8,204	56	±	7
HeLa	1.1%	±	0.3%	9,726	±	2,962	8	±	2
Detection with anti-HER2 DARPin[5]									
BT-474	100.0%			870000			693		
HEK293_Halo-HER2, induced	108.3%			942416			750		
HEK293_Halo-HER2, non-induced	6.9%			60237			48		
HeLa	1.8%			15445			12		

[1] Histograms of the flow cytometry data for the HER2 affibody are shown in Supplementary Figure 2; the histograms for BT474 and HeLa cells measured using the anti-HER2 DARPin-GFP fusion have been previously published[4].
[2] Calculated from the known receptor number for BT-474 cells[5].
[3] Estimating a cell as a sphere with a radius of 10 µm.
[4] Measured by using the anti-HER2 affibody ZHER2 (ref. 6).
[5] Measured by using an anti-HER2 DARPin-GFP fusion[4].

Supplementary Table 2: Diffusion and interaction of HER2 as quantified by SMLM.

		un-treat. (n=3)	OL4G (n=7)	GL4G (n=6)	6L46 (n=8)	PZB (n=5)	TZB (n=3)	6L1G (n=5)
Loc. Density [μm^{-2}]	Median	0.55	0.35	0.63	0.44	0.52	0.56	0.40
	IQR	0.02	0.19	0.14	0.28	0.48	0.17	0.15
	Mean	0.55	0.43	0.59	0.46	0.58	0.60	0.45
	SEM	0.01	0.08	0.04	0.06	0.13	0.07	0.07
Imm. Fraction [%]	Median	5.76	4.16	18.47	15.82	11.87	15.69	60.13
	IQR	2.75	4.21	15.53	6.47	10.58	5.06	15.50
	Mean	5.64	6.03	18.84	15.01	15.19	15.10	56.92
	SEM	1.06	1.92	3.14	1.75	4.30	1.88	5.10
Diff. Coeff [μm^2/s]	Median	0.22	0.19	0.14	0.12	0.12	0.14	0.06
	IQR	0.02	0.03	0.02	0.02	0.02	0.03	0.01
	Mean	0.21	0.20	0.14	0.12	0.13	0.14	0.06
	SEM	0.01	0.01	0.01	0.00	0.01	0.01	0.00
Mobile Dual-lab. Fraction [%]	Median	0.02	0.00	0.34	0.69	0.31	0.25	1.04
	IQR	0.07	0.03	0.45	1.01	0.79	0.27	1.04
	Mean	0.04	0.01	0.37	0.91	0.47	0.25	1.23
	SEM	0.03	0.01	0.11	0.26	0.23	0.08	0.32
Immobile Dual-lab. Fraction [%]	Median	0.00	0.00	0.28	0.04	0.07	0.37	5.90
	IQR	0.02	0.00	0.56	0.20	0.55	0.86	2.99
	Mean	0.01	0.06	0.38	0.12	0.43	0.43	5.44
	SEM	0.01	0.06	0.13	0.06	0.38	0.38	1.15
Confined Frac-tion [%]	Median	4.35	3.13	17.34	11.79	14.67	7.75	57.35
	IQR	1.11	2.65	7.02	6.54	4.47	3.33	5.62
	Mean	3.95	3.59	18.41	13.33	14.71	6.58	60.43
	SEM	0.47	1.03	2.49	1.79	1.36	1.41	2.78
Conf. Trans. Prob. [%]	Median	0.75	0.82	2.27	2.85	2.69	1.66	10.75
	IQR	1.13	0.44	0.88	0.88	0.72	0.57	3.92
	Mean	1.01	0.94	2.25	2.88	2.65	1.66	11.08
	SEM	0.45	0.09	0.32	0.24	0.28	0.22	0.92
Mob. Trans. Prob. [%]	Median	5.96	10.09	3.92	8.55	7.29	6.71	3.56
	IQR	1.58	6.68	1.97	3.81	2.67	1.03	1.84
	Mean	6.38	11.29	4.72	9.82	8.30	6.65	3.57
	SEM	0.64	1.66	0.83	1.22	1.47	0.40	0.51
Mobility Half-Time [s]	Median	2.93	2.70	0.99	0.77	0.81	1.33	0.20
	IQR	3.40	1.05	0.40	0.27	0.26	0.48	0.07
	Mean	3.26	2.48	1.10	0.80	0.88	1.37	0.19
	SEM	1.32	0.22	0.19	0.07	0.12	0.18	0.02
Confinement Half-Time [s]	Median	0.36	0.21	0.56	0.25	0.29	0.32	0.61
	IQR	0.08	0.13	0.23	0.09	0.08	0.05	0.35
	Mean	0.34	0.21	0.52	0.23	0.28	0.32	0.68
	SEM	0.03	0.03	0.07	0.02	0.04	0.02	0.12

IQR, interquartile range; SEM, standard error of the mean. *n* denotes the number of cells analyzed per condition.

Supplementary methods

Confocal single-molecule fluorescence spectroscopy

Confocal single-molecule fluorescence measurements were conducted on a customized MicroTime 200 (PicoQuant) based using an Olympus IX71 microscope body as previously described[7]. In brief, fluorescent dyes were excited alternatingly[8] by a 20-MHz supercontinuum laser (SC-450-4, Fianium, with a 520/15 band-pass filter, Chroma Technology) and a 635-nm pulsed laser (PicoQuant) at 5 µW of excitation power each, as measured at the back aperture of the objective. The light was focused into the sample by an UplanApo 60×/1.20-W objective (Olympus). SPCM-AQR-15 single-photon avalanche diodes (PerkinElmer) were used to detect photons from each channel, and their arrival times were recorded in four channels of a HydraHarp 400 counting module (PicoQuant) with a resolution of 16 ps. A piezo stage combination (P-733.2 and PIFOC, Physik Instrumente GmbH), to which the objective was mounted, enabled 3D scans. After performing an initial x-y scan to obtain cell outlines, time traces were recorded at a fixed point in the apical (upper) cell membrane for 180 s. Data processing and analyis (binning: 100 ms for all samples) were done using Fretica, a Wolfram Symbolic Transfer Protocol add-on for for Mathematica (Wolfram Research) (https://schuler.bioc.uzh.ch/programs/). The first 100 s, where the background signal is not constant due to bleaching, were excluded from burst detection. A binning time of 1 ms, a burst threshold of 10 photons per bin, and 20 and 10^6 photons as lower and higher bounds for the total number of photons in a burst, respectively, were used for burst identification.

Flow cytometry

Subconfluent cells were harvested using trypsin. 1×10^6 cells (as determined using a CASY TT cell counter, OLS OMNI Life Science) were washed once by resuspension in 1 ml Dulbecco's phosphate buffered saline (DPBS) and subsequent centrifugation at 800 × g for 1 min. Cells were then resuspended in 1 ml DPBS supplemented with 1% bovine serum albumin and 50 mM sodium azide and (PBSBA), which furthermore contained 500 ng ml^{-1} of 4',6-diamidino-2-phenylindole (DAPI). All samples were then split up into two aliquots of 500 µl each. To one of the aliquots, 6 µM of non-labeled affibody ZHER2 was added, and the samples incubated for 20 min at room temperature. Subsequently, ZHER2 conjugated to DY-647-P1 was added to a concentration of 40 nM, and the samples incubated for further 20 min. Finally, the samples were washed three times in PBSBA as described above and transferred to FACS tubes (BD)

prior to measurement on a LSR Fortessa II (BD) flow cytometer. Data were analyzed in FlowJo 10.7.1 (FlowJo, LLC).

Supplementary references

1. Vogelsang, J. *et al.* A reducing and oxidizing system minimizes photobleaching and blinking of fluorescent dyes. *Angew. Chem. Int. Ed.* **47**, 5465-5469 (2008).
2. Sprague, B.L., Pego, R.L., Stavreva, D.A. & McNally, J.G. Analysis of binding reactions by fluorescence recovery after photobleaching. *Biophys. J.* **86**, 3473-3495 (2004).
3. Ester, M., Kriegel, H.-P., Sander, J. & Xu, X. in *Proceedings of the Second International Conference on Knowledge Discovery and Data Mining* 226–231 (AAAI Press, Portland, Oregon; 1996).
4. Hansen, S. et al. Design and applications of a clamp for Green Fluo-rescent Protein with picomolar affinity. *Sci. Rep.* **7**, 16292 (2017).
5. Zahnd, C. *et al.* Efficient tumor targeting with high-affinity designed ankyrin repeat proteins: effects of affinity and molecular size. *Cancer Res.* **70**, 1595-1605 (2010).
6. Eigenbrot, C., Ultsch, M., Dubnovitsky, A., Abrahmsén, L. & Härd, T. Structural basis for high-affinity HER2 receptor binding by an engineered protein. *Proc. Natl. Acad. Sci. U. S. A.* **107**, 15039-15044 (2010).
7. König, I. *et al.* Single-molecule spectroscopy of protein conformational dynamics in live eukaryotic cells. *Nat. Methods* **12**, 773-779 (2015).
8. Müller, B.K., Zaychikov, E., Bräuchle, C. & Lamb, D.C. Pulsed interleaved excitation. *Biophys. J.* **89**, 3508-3522 (2005).
